# A review of the sources and pharmacological research of morroniside

**DOI:** 10.3389/fphar.2024.1423062

**Published:** 2024-09-04

**Authors:** Pengliang Shi, Bingqing Zheng, Shiyao Zhang, Qingmei Guo

**Affiliations:** School of Pharmacy, Shandong University of Traditional Chinese Medicine, Jinan, China

**Keywords:** morroniside, sources, pharmacology, distribution, detection

## Abstract

**Introduction:**

Morroniside (Mor) is a bioactive compound found in *Corni Fructus* (*CF*) [Cornaceae; *Cornus officinalis* Siebold & Zucc.], which has been used as medicine and food in China, Korea, and Japan for over 2,000 years. This review summarizes recent progress on Mor, specifically focusing on its distribution, isolation, detection, and various pharmacological effects.

**Methods:**

A literature survey on Mor was conducted using electronic databases such as PubMed, ScienceDirect, CNKI, and Google Scholar. After removing TCM prescription-related standards, medicinal herb processing-related research, and other irrelevant works of literature, we obtained relevant information on Mor’s biological and pharmacological properties.

**Results:**

The main conclusions are as follows: Mor is widely distributed in the plant kingdom; the methods for extracting and isolating Mor are well established; and the technology for detecting it is accurate. Mor exhibits numerous pharmacological effects. Along with *CF*, Mor has shown renoprotective effects against diabetes, hepatoprotective effects against diabetes, triptolide, and nonalcoholic steatohepatitis, and boneprotective effects against osteoporosis and osteoarthritis. In addition, researchers have also explored other pharmacological effects of Mor, including neuroprotective effects against focal cerebral ischemia, spinal cord injury, and Alzheimer’s disease; cardioprotective effects against acute myocardial infarction; protection of the digestive system from gastritis, inflammatory bowel disease, and colitis; protection of the skin by promoting hair growth, wound healing, and flap survival; and protection of the lungs from acute lung injury and pulmonary fibrosis. Moreover, Mor has anti-obesity effects, anti-inflammatory effects in the eye, and improves follicular development.

**Discussion:**

Overall, this review provides a comprehensive understanding of the pharmacological effects of Mor, from which the limitations of the current research can be understood, which will help facilitate future research.

## 1 Introduction

*Corni Fructus* (*CF*) was first recorded in Shen Nong Materia Medica and extensively used as a medicine and food in China, Korea, and Japan. Its main functional indications are replenishing the liver and kidney and arresting the loss of essence (Chinese Pharmacopoeia, 2020). Liu Wei Di Huang Wan (六味地黄丸) (Ge et al., 2018) and Jin Gui Shen Qi Wan (金匮肾气丸) (Xu and Cao, 2015) are the famous representatives of replenishing the liver and kidney.

**FIGURE 1:**
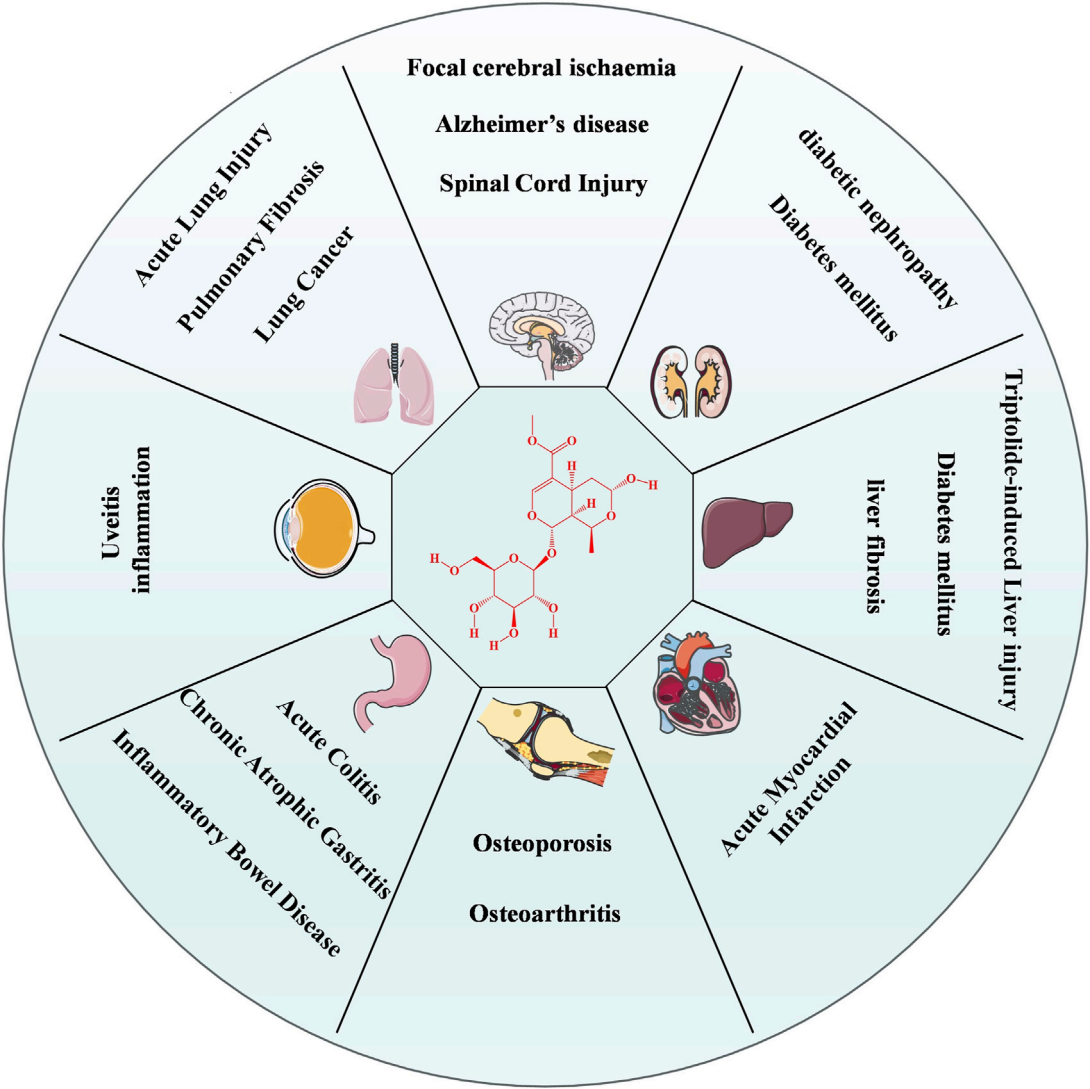
Pharmacological effects of Mor.

Morroniside (Mor), an iridoid glycoside (Figure 1), is the primary bioactive and representative ingredient in *CF*. With the continuous expansion of the pharmacological effects of *CF*, Mor has attracted attention. The multiple pharmacological effects, including neuroprotective, bone-protective, cardioprotective, renoprotective, and hepatoprotective effects, suggest that Mor has considerable potential value in the prevention and treatment of diseases such as focal cerebral ischemia, spinal cord injury, Alzheimer’s disease, osteoporosis, osteoarthritis, acute myocardial infarction, and diabetes. This review provides the latest and most comprehensive data on Mor’s extraction, detection, and pharmacology, which will help facilitate further research and provide a reference resource for its clinical application.

## 2 Sources of Mor

### 2.1 Distribution of Mor

The compound Mor is found in a wide variety of plants. To date, 17 plant species have been reported to contain Mor. Detailed information and images of these plants can be found in Table 1. These species include *Sambucus williamsii* (Liang et al., 2011), *Chione venosa* (Lendl et al., 2005), *Adina pilulifera* (Xue et al., 2007), *Lonicera macranthoides* (Sun et al., 2011), *Sarracenia purpurea* (Cieniak et al., 2015), *Mussaenda luteola* (Mohamed et al., 2016), *L. japonic*a (Cai et al., 2019), *Angelica gigas*, *Glycyrrhiza uralensis*, *G. glabra*, *G. inflata*, *Schisandra chinensis* (Ahn et al., 2020), *Gentiana straminea* (Zhou et al., 2021), *Patrinia scabra* (Ma et al., 2015), *Caulophyllum robustum* (Li et al., 2015), and *Gentiana olivieri* Griseb (Maituoheti et al., 2023). They belong to class Angiospermae and are broadly categorized into Apiaceae, Berberidaceae, Caprifoliaceae, Fabaceae, Gentianaceae, Rubiaceae, Sarraceniaceae, and Viburnaceae. Mor has a large reserve in the plant kingdom; therefore, developing and utilizing these plants would be beneficial. In addition, researchers discovered that the *3-hydroxy-3-methylglutaryl-CoA synthase (HMGS)* gene plays a crucial role in the synthesis of Mor in Cornus officinalis (Zhang et al., 2023) and that the *C. officinalis geranyl pyrophosphate synthase (CoGPPS)* is another critical gene involved in the biosynthesis of Mor (Chen J. et al., 2024). This discovery offers valuable insight into the genetic mechanism responsible for the production of Mor in this plant.

**TABLE 1:** Plant species information and their respective photos (representative pictures provided by https://www.plantplus.cn/cn).

Number	Botanical name	Species	Representative picture	Reference
1	*Angelica gigas*	[Apiaceae; *Angelica gigas* Nakai]	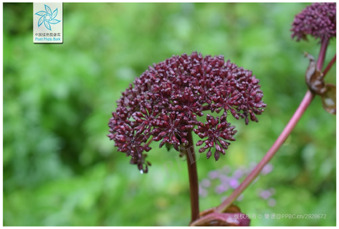	Ahn et al. (2020)
2	*Caulophyllum robustum*	[Berberidaceae; *Caulophyllum robustum* Maxim]	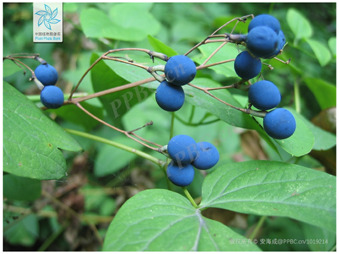	Li et al. (2015)
3	*Patrinia scabra*	[Caprifoliaceae; *Patrinia scabra* Bunge]	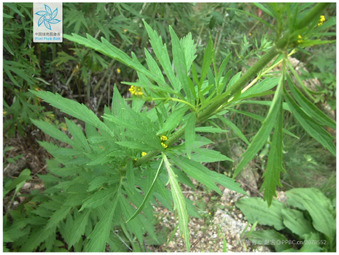	Ma et al. (2015)
4	*Lonicera macranthoides*	[Caprifoliaceae; *Lonicera macranthoides* Hand.-Mazz.]	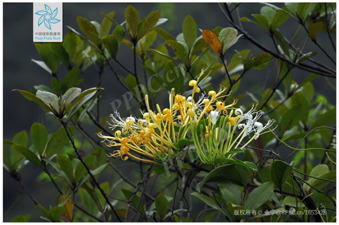	Sun et al. (2011)
5	*Lonicera japonic*a	[Caprifoliaceae; *Lonicera japonica* Thunb.]	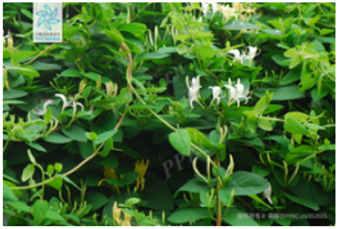	Cai et al. (2019)
6	*Cornus officinalis*	[Cornaceae; *Cornus officinalis* Siebold and Zucc.]	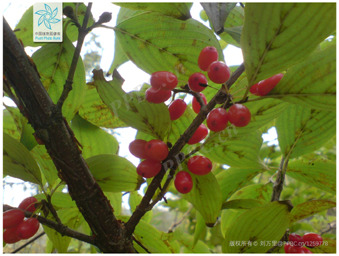	Li et al. (2006)
7	*Glycyrrhiza uralensis*	[Fabaceae; *Glycyrrhiza uralensis* Fisch. ex DC.]	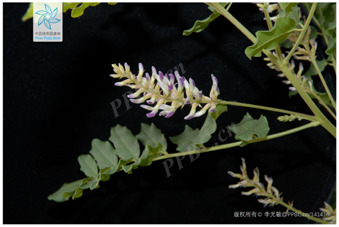	Ahn et al. (2020)
8	*Glycyrrhiza glabra*	[Fabaceae; *Glycyrrhiza glabra* L.]	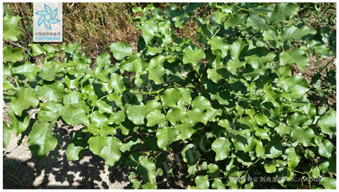	Ahn et al. (2020)
9	*Glycyrrhiza inflata*	[Fabaceae; *Glycyrrhiza inflata* Batalin]	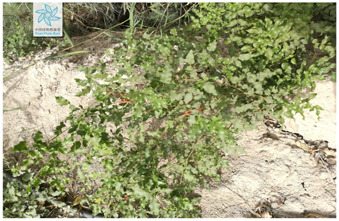	Ahn et al. (2020)
10	*Gentiana straminea*	[Gentianaceae; *Gentiana straminea* Maxim.]	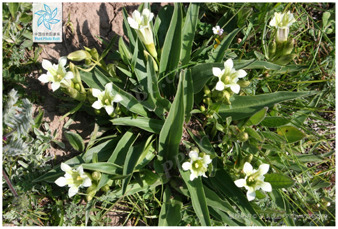	Zhou et al. (2021)
11	*Adina pilulifera*	[Rubiaceae; *Adina pilulifera* (Lam.) Franch. ex Drake]	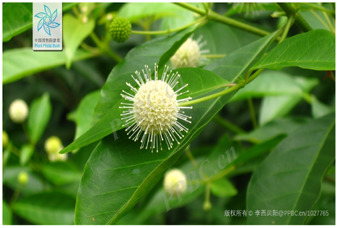	Xue et al. (2007)
12	*Chione venosa*	[Rubiaceae; *Chione venosa* (Sw.) Urb.]	—	(Lendl et al., 2005)
13	*Mussaenda luteola*	[Rubiaceae; *Pentas lanceolata* (Forssk.) Deflers]	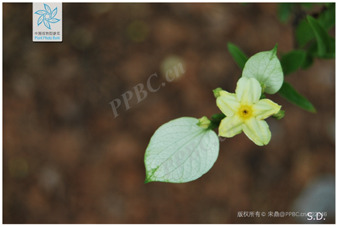	Mohamed et al. (2016)
14	*Sarracenia purpurea*	[Rubiaceae; *Sarracenia purpurea* L.]	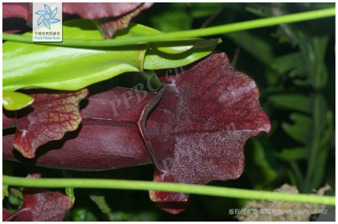	Cieniak et al. (2015)
15	*Schisandra chinensis*	[Rubiaceae; *Schisandra chinensis* (Turcz.) Baill.]	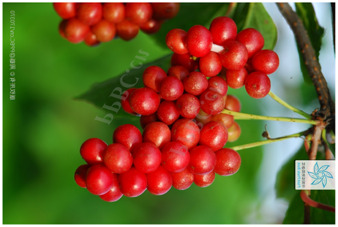	Ahn et al. (2020)
16	*Sambucus williamsii*	[Viburnaceae; *Sambucus williamsii* Hance]	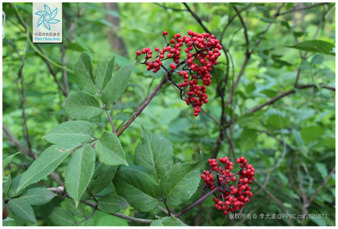	Liang et al. (2011)
17	*Sambucus williamsii Hance* var. *miquelii* (The name has been revised to Sambucus sibirica)	[Viburnaceae; *Sambucus sibirica* Nakai]	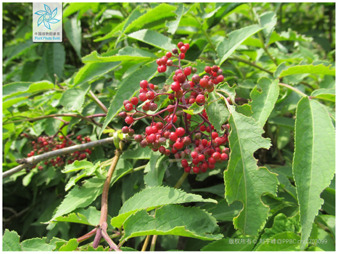	Mu et al. (2024)
18	*Gentiana olivieri*	[Gentianaceae; *Gentiana olivieri* Griseb.]	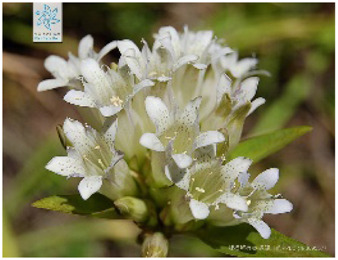	Maituoheti et al. (2023)

### 2.2 Isolation and purification of Mor

Mor is mainly extracted and separated from *CF*, and this article reviews the techniques and simpler steps used in purification. Isolation and purification of Mor are performed as follows: CF is extracted with hot water and precipitated by adding alcohol (four times the amount of water). Subsequently, the extract is concentrated using a rotary evaporator and isolated using AB-8 macroporous resin. Afterward, 30% ethanol eluent is pooled and passed through silica gel with detection using thin-layer chromatography; then, the mixture is purified by high-performance liquid chromatography (HPLC) to 98% (Li et al., 2006). In a simplified process, Mor is obtained from CF in a two-step separation process. In brief, a fraction of 40% ethanol is collected using a macroporous absorption resin column, following ultrasonic extraction with 50% methanol. Subsequently, high-speed countercurrent chromatography (CCC) separation is performed to achieve a final purity of 97.8% (Liu et al., 2009). Another extraction method consists of refluxing with 75% ethanol. Afterward, 50% aqueous ethanol eluent is concentrated after the water elution becomes colorless using D101 macroporous resin. The mixture is subjected to CCC separation to achieve a final purity of 99.1% (Liang et al., 2013). Moreover, *CF* was crushed and extracted with 70% ethanol. The resulting extracts were filtered, evaporated, and freeze-dried. The fraction was obtained after being subjected to Diaion HP-20 chromatography using 30% ethanol and was further separated using a C18 MPLC (Oh et al., 2024). With the rapid progress in technology, particularly in the development of polymer materials, the isolation and purification of Mor from plants are anticipated to become more convenient, efficient, and eco-friendly. This encourages greater exploration and study of Mor, increasing possibilities for its utilization.

### 2.3 Detection of Mor

The detection of Mor is essential for the quality control of *CF*, and upgrading detection technology makes the detection method more accurate, simple, and convenient. The detailed detection methods are summarized in Table 2 Using micellar electrokinetic capillary chromatography (MEKC), Mor has been isolated from eight other components in *CF* (Wang et al., 2003) and Liu Wei Di Huang Wan (Zhao et al., 2007). Additionally, Mor has been detected using the diode array detector (DAD) and variable wavelength detector (VWD) at 218 nm (Du et al., 2008), evaporative light-scattering detector (ESLD), and electrospray ionization mass spectrometry (ESI-MS) (Ye et al., 2009). Paper spray mass spectrometry (PS-MS) (Guo et al., 2017) and near-infrared spectroscopy (NIRS) (Gong et al., 2015) have been applied to evaluate *CF* quality. Pre-treatment methods have been improved to simultaneously detect as many iridoid glycosides as possible. Specifically, ionic liquid-based vortex-assisted matrix solid-phase dispersion (Du et al., 2018), molecularly imprinted solid-phase extraction (Ji et al., 2018) have been used to detect iridoid glycosides in *CF* and Liu Wei Di Huang Wan. The specialization of pre-treatment technology and the diversification of detection technology allow Mor to be analyzed qualitatively and quantitatively more rapidly and accurately. Moreover, a qualitative analysis of Mor was conducted among 134 compounds in the Shandong Yangxin capsule using ultra-high-performance liquid chromatography–Fourier-transform ion cyclotron resonance mass spectrometry (UPLC-FT-ICR-MS) (Fu et al., 2024). Another study demonstrated that ultra-performance liquid chromatography coupled with linear quadrupole ion trap-orbitrap mass spectrometry (UHPLC-LTQ-Orbitrap-MS) was able to identify 130 components of CF, including Mor (Sun et al., 2023).

**TABLE 2:** Different detection methods for Mor.

Num	Detection method	Specific setting condition	Reference
1	MEKC	Buffer: 10 mM NaH_2_PO_4_, 5 mM Na_2_B_4_O_7_, 120 mM SDS, and 5% (v/v) methanol with pH 6.3 to 7.8. Capillary: 50 cm × 75 μm; voltage 12.5 Kv; cartridge temperature: 25.0°C; with 214 nm	Wang et al. (2003)
2	MEKC	Buffer: 0.2 M boric acid, 0.02 M SDS, pH 10.5, with various volume percentage of acetonitrile, fused silica capillary: 60 cm × 75 μm; voltage, 14 kV; with 240 nm	Zhao et al. (2007)
3	DAD	C18 column (250 mm × 4.6 mm, 5 μm) with 240 nm or 218 nm, and no signal with 280 nm	Du et al. (2008)
4	DAD	C18 column (250 mm × 4.6 mm, 5 μm) with 254 nm or 238 nm	Ye et al. (2009)
5	ELSD	C18 column (250 mm × 4.6 mm, 5 μm) with Drift tube temperature: 40°C Nebulization air: 3.5 bar	Ye et al. (2009)
6	ESI-MS	C18 column (250 mm × 4.6 mm, 5 μm) with drying gas 10.0 L/min; temperature 350°C; nebulizer gas 30 psi; spray voltage 3.5 kV; and 429 [M + Na]^+^, 227 [M + H-glu]^+^ 405 [M−H]^−^, 451 [M + HCOO]^−^	Ye et al. (2009)
7	HPLC–MS/MS	C18 column (100 mm × 3.0 mm, 3.5 μm) with spray voltage 4 kV (+), 3.6 kV (−); source temperature 100°C; desolvation temperature 350°C; nebulizer gas 40 psi; and precursor ion: 451 [M + HCOO], product ion: 243 and 179	Cai et al. (2013)
8	PS-MS	A triangular piece of chromatography paper, 10 mm (height) × 5 mm (base) was set 4–6 mm from the inlet of the MS with 445.10 [M + K]^+^ and 429.15 [M + Na]^+^	Guo et al. (2017)
9	UHPLC-LTQ-Orbitrap-MS	C18 column (2.1 × 100 mm, 1.7 μm) with ion spray voltage 3.5 kV; capillary temperature 350°C; capillary voltage, −27 V; tube lens voltage, −198 V; and sheath gas 40; auxiliary gas (He) 10; and precursor ion: 451.14343 [M + HCOO]^−^ fragment ions: 405.13800, 373.11359, 243.08623, 179.05540, 255.07579	Sun et al. (2023)
10	UPLC-FT-ICR-MS	C18 column (150 × 2.1 mm, 1.8 μm) with capillary voltage 4.5 kV; dry gas temperature 200°C dry gas flow rate 8 L/min; ion accumulation time 0.15 s; flight time 0.6 m; nebulizer gas 4 bar; collision energy 10–30 eV; and measured mass: 405.14024 MS/MS: 373.1124, 243.0877, 155.0347, 141.0570, 123.0451	Fu et al. (2024)

MEKC effectively combines the advantages of chromatography and electrophoresis, making it widely used in protein and peptide separations. In recent years, the separation and detection of small molecules have become a new research direction, and its coupling with MS also provides the possibility for the detection of Mor. The convenience and accuracy of the HPLC system, in combination with DAD or ELSD, make it the most widely used method for qualitative and quantitative analyses of Mor. UPLC-FT-ICR-MS and UHPLC-LTQ-Orbitrap-MS can be used for the qualitative analysis of Mor very accurately, which provides strong technical support for the expansion of medicinal plant resources of Mor and the serum pharmacology and pharmacokinetics of prescriptions containing Mor.

## 3 Pharmacological effects of Mor

### 3.1 Protective effects of Mor on the nervous system

**FIGURE 2:**
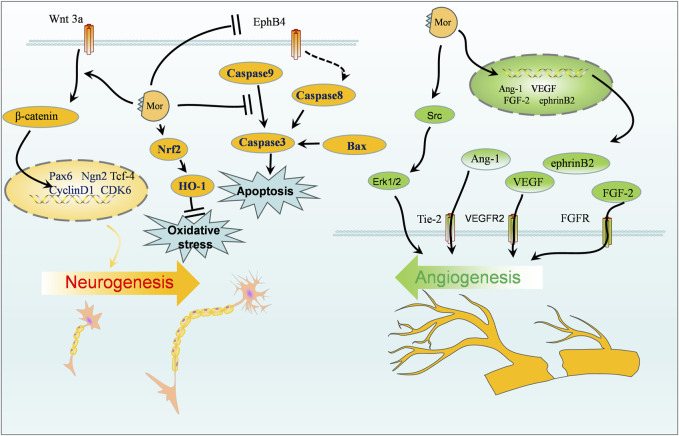
Protection of Mor against focal cerebral ischemia.

**FIGURE 3:**
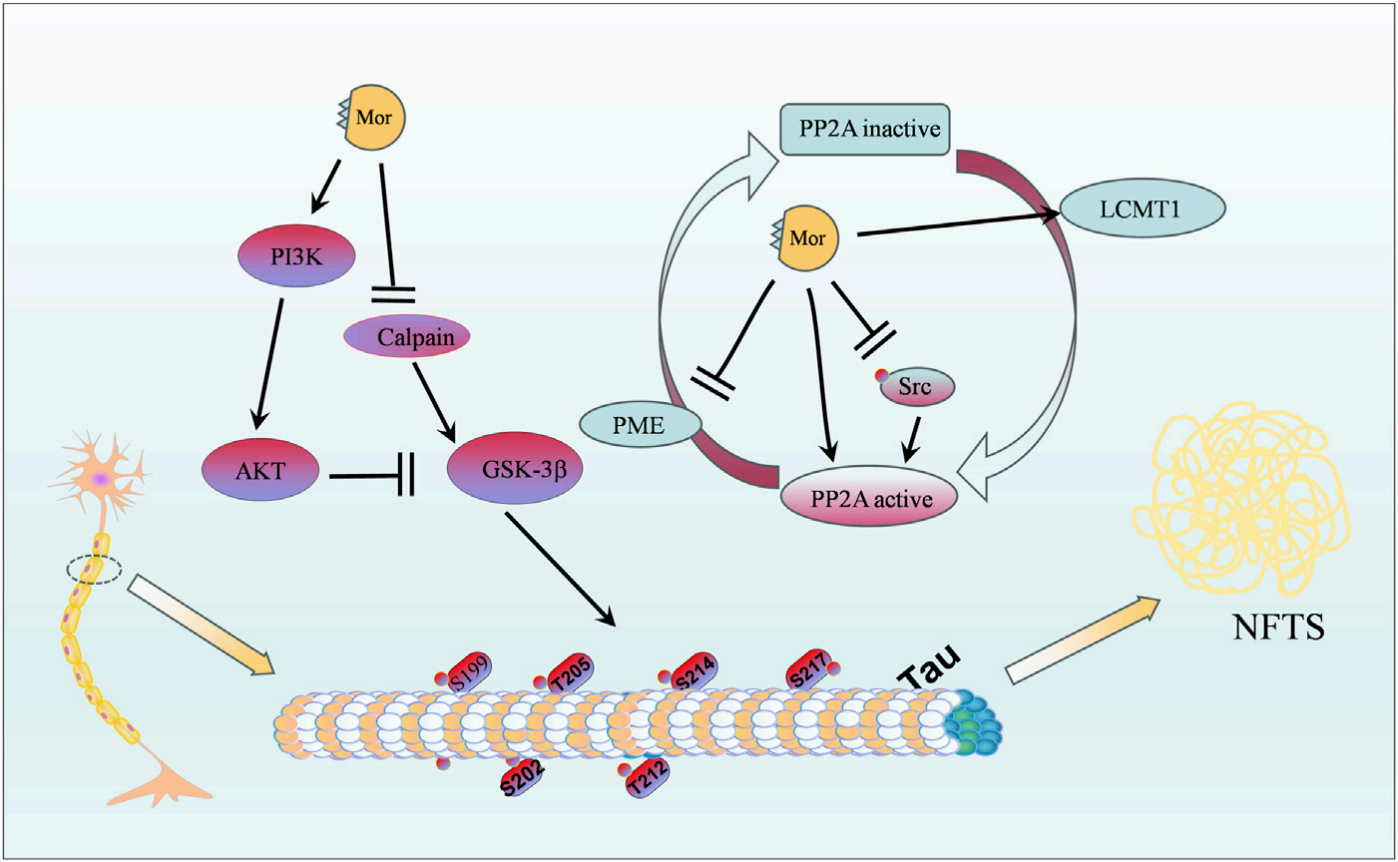
Protective effects of Mor against Alzheimer’s disease.

The protective effect of Mor on the nervous system can be classified into four categories: protection against focal cerebral ischemia, spinal cord injury, Alzheimer’s disease, and neuroprotective effects. The vital targets and putative pathways of Mor on the nervous system are summarized in Figures 2, 3, detailed pharmacological effects was listed in Table 3, and therelevant mechanisms are described as follows.

**TABLE 3:** Neuroprotective activity.

Cell or animal model and method	Application	Dose	Molecular mechanism	Reference
H_2_O_2_-induced SH-SY5Y cells	*In vitro*	1, 10, and 100 μM	Oxidative stress: ROS, lipid peroxidation ↓ Apoptosis: caspase-3, caspase-9↓ Inflammation: Bcl-2 ↑, Bax →	Wang et al. (2008)
H_2_O_2_-induced SH-SY5Y cells	*In vitro*	1, 10, and 100 μM	SOD↑, LDH↓ intracellular accumulation of Ca^2+^ ↓ MMP↓	Wang et al. (2009)
H_2_O_2_-induced SK-N-SH cells	*In vitro*	1, 10, and 100 μM	Oxidative stress: ROS, lipid peroxidation ↓ Apoptosis: caspase-3↓; Bcl-2 ↑, Bax↓	Zhang et al. (2017)
H_2_O_2_- or Aβ1-42-induced rat PC12 cells	*In vivo*	10, 50, and 100 μM	Apoptotic: Bcl-2↑, Bax ↓, cytochrome C↓, cleaved caspase-3↓ JNK↓, p38 MAPK↓	Chen et al. (2018)
Depression model rat	*In vivo*	200 mg/kg	Inflammation: IL-1β↓, TNF-α↓, NF-κBp65↓	Mo (2020)
MCAO rat	*In vivo*	30, 90, and 270 mg/kg	Oxidative stress: MDA ↓, GSH ↓, SOD ↓ Apoptosis: caspase-3 ↓ BBB integrity ↑	Wang et al. (2010a)
MCAO rat	*In vivo*	30, 90, and 270 mg/kg	MMP2, MMP9 ↓; caspase-3 ↓; Bcl-2/Bax ↑	Zeng et al. (2018)
MCAO rat	*In vivo*	30, 90, and 270 mg/kg	BBB integrity: MMP2, MMP9 ↓, IL-1β↓ EPCs proliferation: newly born vascular endothelial cells ↑, Ang-1, Tie-2, NRP-1, FGF-2, VEGF, vWF + vessels ↑	Sun et al. (2014a)
MCAO rat	*In vivo*	270 mg/kg	Angiogenesis: VEGFR2, ephrinB2, Erk1/2, and Src ↑	Liu et al. (2016)
MCAO rat	*In vivo*	30, 90, and 270 mg/kg	Endogenous neural stem cells ↑ Wnt 3a, β-catenin, and Tcf-4 ↑ Pax6, Ngn2 ↑	Sun et al. (2014b)
OGD/R-induced HT-22 cells	*In vitro*	5, 10, and 20 μM	Oxidative stress: Nrf2, HO-1 ↑ Inflammation: Bcl-2 ↑, Bax ↓ Apoptosis: cleaved Caspase-3 ↓, cleaved caspase-9 ↓	Zhang et al. (2022a)
Microglial cells	*In vitro*	0.1, 1 mM, and 200 μM	M2 polarization ↑: Agr1, C206, IL-4, and IL-10 ↑ M2 polarization ↑: p38↓	Liu et al. (2021b)
MCAO rat	*In vivo*	300, 500, and 1,000 μg/5 μL	IL-10 ↑	Liu et al. (2021b)
H2O2-induced OLN-93 cells	*In vitro*	200 μM	Oxidative stress: SOD2, iNOS↓ Inflammation: Bcl-2 ↑, Bax ↓ Apoptosis: cleaved caspase-3 ↓	Li et al. (2021)
SCI-injured rats	*In vivo*	180 mg/kg	Apoptosis: caspase-3 ↓ Oxidative stress: H2O2↓, MDA↓, SOD↑, CAT↑, GSH-PX↑ 8-OHDG↓,3-NT↓	Duan et al. (2021)
Okadaic acid-treated SK-N-SH cells	*In vitro*	50, 100, and 200 μM	PP2A↑; Ser199/202↓, Thr205↓, Thr 212↓, Ser214↓, Thr217↓; tau 5↑	Yang et al. (2016)
PP2Ac siRNA-transfected HEK293 cells	*In vitro*	50, 100, and 200 μM	PME-1/LCMT-1↓; the phosphorylation of Src at Tyr416↓	Yang et al. (2016)
N2a cells	*In vitro*	240 μM	GSK-3β↓, PP2A↑	Guo et al. (2022)
Rat-ligated the L5/L6 spinal nerves	*In vivo*	300 mg/kg	Mechanical anti-allodynic and thermal anti-hyperalgesic effects↑	Meng et al. (2017)

#### 3.1.1 Protection of Mor against focal cerebral ischemia

Cerebral ischemia can result in death or permanent disability; the limited blood flow to the brain cuts off oxygen and other nutrients, causing cerebral ischemia (Girnar and Mahajan, 2021). Cerebral ischemia therapy is closely linked to angiogenesis, neurogenesis, blood–brain barrier (BBB), and cerebral blood flow (Ahad et al., 2020; Herpich and Rincon, 2020). Animal models of transient middle cerebral artery occlusion (MCAO) and permanent occlusion of extracranial vessels have been created to simulate cerebral ischemia conditions.

In MCAO rats, Mor improved neurobehavioral scores (Zea-Longa, Ludmila Belayer, and prehensile traction), reduced brain infarction volume, minimized oxidative stress by regulating MDA, glutathione (GSH), and superoxide dismutase (SOD), and reduced apoptosis by decreasing MMP2, MMP9, and caspase-3 while increasing the ratio of Bcl-2/Bax (Wang W. et al., 2010; Zeng et al., 2018).

Angiogenesis relies on the proliferation of endothelial progenitor cells (EPCs) (Wang et al., 2021). In MCAO, Mor increased vWF^+^ vessels and CD34^+^ cells, indicating an increase of new blood vessels in the brain, and angiogenic promoters (Ang-1, Tie-2, NRP-1, FGF-2, HGF, and VEGF) elevated EPC proliferation (Sun et al., 2014a; Wei et al., 2016; Liu et al., 2019). Previous studies on the long-term effects of Mor on angiogenesis in MCAO have shown that new vessels were generated after 14 days in the peri-infarcted cortex, and angiogenesis-related proteins, such as vascular endothelial growth factor receptor 2 (VEGFR2), ephrin-B2, Erk1/2, and Src, were increased. Additionally, in MCAO, regional cerebral blood flow dynamics and the number of vessels of the leptomeningeal anastomoses were improved by Mor, which indicated that the new vessels improved microvascular circulation (Liu et al., 2016).

Neurogenesis plays another important role in ischemia stroke treatment (Ruan et al., 2015). In MCAO rats, Ludmila Belayer demonstrated that the neurological function was improved by Mor, as well as the increase in endogenous neural stem cells marked with Ki-67 and nestin (a predominant protein marker for neural stem and progenitor cells) in the ischemic ipsilateral dorsolateral corner of the subventricular zone, the unilateral ventricle wall, and peri-infarct cortex. Furthermore, Mor increased the expression of Wnt3a, β-catenin, and Tcf-4, along with activating the downstream transcription factors Pax6 and Ngn2, but did not influence Tbr2 expression (Sun et al., 2014b). In addition, Mor promoted the proliferation of neural stem cells from oxygen-glucose deficiency and differentiation into neurons, as marked by Map2 and GFAP; after transfected with shRNA-EphB4, Mor still promoted the proliferation and differentiation with the weakened efforts (Sun et al., 2019). The accumulation of cyclin D1 caused by cerebral ischemia promotes programmed cell death by activating CDKs (Wen et al., 2005); Mor downregulated cyclin D1 and CDK6 to protect the brain (Liu et al., 2013). In HT-22 cells deprived of transitory oxygen and glucose, Mor increased viability and suppressed oxidative stress by reducing ROS and MDA while increasing SOD and GSH. Furthermore, Mor upregulated Nrf2, HO-1, and Bcl-2, downregulated Bax, cleaved caspase-3, and cleaved caspase-9 (Zhang L. et al., 2022). Therefore, Mor promotes neurogenesis to protect cerebral ischemia, which primarily promotes neuro differentiation, minimizes programmed cell death, reduces oxidative stress, and inhibits apoptosis.

The transcriptomic analysis of the brain following ischemia revealed that the microglia contributed 75% of the differentially expressed genes, which were primarily related to activity, differentiation, metastasis, and inflammation (Khan et al., 2017). Mor induced M2 polarization in primary microglial cells by increasing the expression of Agr1, C206, IL-4, and IL-10. Furthermore, Mor only affected IL-10 expression in microglial cells compared with astrocytes and neural cells. In another study, among various inhibitors, only p38 inhibitors reduced IL-10 expression in MCAO mice. At the same time, intracerebroventricular injection of Mor increased IL-10 expression in the cortical penumbra area, which reduced the infarction size reversed by exendin(9–39), suggesting that Mor is a GLP-1R agonist. Mor protects microglial cells by inducing M2 polarization and IL-10 expression in M2 microglia, possibly mediated by the cAMP/PKA/p38β pathway (Liu et al., 2021b).

#### 3.1.2 Protection of Mor against spinal cord injury

Spinal cord injury (SCI) results in permanent neurological impairment with nearly no effective treatment, leading to severe lifelong disabilities and a significant burden to individuals, families, and society (Sachdeva et al., 2018). In OLN-93 cells (a cell line of oligodendrocytes) induced by H_2_O_2_, Mor diminished oxidative stress by attenuating ROS and MDA and increased mitochondrial membrane potential (MMP) by suppressing SOD2 and iNOS. Mor resisted apoptosis by upregulating Bcl-2 and downregulating cleaved caspase-3 and Bax. In addition, LY294002, an inhibitor of the PI3K/AKT pathway, inhibits the protective effect of Mor described above; therefore, Mor may protect SCI against oxidative stress and apoptosis via the PI3K/AKT signaling pathway (Li et al., 2021).

In another study, Mor has been shown to significantly improve the locomotor function of SCI rats, according to the Basso, Beattie, and Bresnahan locomotor rating scale, reduce the lesion area, improve the preservation of myelin, and alleviate motor neuron loss. In addition, Mor protected nerve cells from apoptosis by reducing caspase-3 expression and extenuated oxidative stress by increasing antioxidant enzymes (SOD, CAT, and GSH) and inhibiting oxygen free radicals (H_2_O_2_, MDA, 8-OHDG, and 3-NT) (Duan et al., 2021). According to RNA sequencing (RNA-seq) results, the specific mechanism is related to Mor’s anti-inflammatory and anti-apoptotic effects in SCI rats (Shi et al., 2022).

#### 3.1.3 Protection of Mor against Alzheimer’s disease

Alzheimer’s disease is a common geriatric disease with considerable individual, social, and economic burden, which is increasingly attracting attention due to the growing elderly population (Author Anonymous, 2021). Studies have demonstrated that the pathogenesis of Alzheimer’s disease is related to abnormal deposition of β-amyloid protein, neurofibrillary tangles (NFTs) caused by tau protein hyperphosphorylation, neuroinflammatory response, mitochondrial dysfunction, and abnormal synaptic transduction function (DeTure and Dickson, 2019). Tau protein hyperphosphorylation in Alzheimer’s disease is attributed to low PP2A expression (Liu et al., 2005).

In P301S mice, a widely used transgenic model of tauopathy, the *CF* extract attenuated tau hyperphosphorylation at Thr205, Ser212, Ser262, Thr231, Ser235, and Ser404 (Yang et al., 2020). In AutoDock and surface plasmon resonance simulation tests, Mor showed high binding free energies and interactions as an inhibitor of acetylcholinesterase, butyrylcholinesterase, and beta-secretase 1 for treating Alzheimer’s disease (Bhakta et al., 2016). Calpain is abnormally activated in NFTs, N2a cells, and P301S mice; Mor inhibited the activity of calpain and glycogen synthase kinase 3β (GSK-3β) and enhanced the activity of PP2A (Guo et al., 2022).

In okadaic acid, a PP2A inhibitor, stimulated SK-N-SH cells, Mor attenuated tau hyperphosphorylation (Ser199/202, Thr205, Thr212, Ser214, and Thr217) by increasing PP2A and tau 5. Importantly, in HEK293 cells where PP2Ac expression was silenced by PP2Ac siRNA, the reduced effect of Mor on tau hyperphosphorylation suggests that Mor directly activates the PP2A protein. Additionally, the ratio of PME-1 (a methylesterase) to LCMT-1 (a leucine carboxyl methyltransferase) was significantly decreased by Mor, which inhibited PP2Ac demethylation. Mor could decrease the phosphorylation of Src, a membrane-associated protein tyrosine kinase that promotes the phosphorylation of PP2Ac at Tyr307 (Yang et al., 2016). Mor also reduced tau protein hyperphosphorylation by activating PP2A or decreasing GSK-3β.

The activation of GLP-1 receptors (GLP-1R) plays protective roles against multiple neurodegenerative disorders, including Alzheimer’s disease and Parkinson’s disease, as well as diabetes mellitus and ischemia (Grieco et al., 2019). In H_2_O_2_-induced microglia N9 and HEK293 cells, the protective effects of Mor were antagonized by exendin(9–39), a selective GLP-1 receptor antagonist, suggesting that Mor acts as a GLP-1R agonist (Meng et al., 2017). In the neuropathic pain rat model established by ligating the L5/L6 spinal nerves, Mor elevated the tolerance of the paw to electronic von Frey filaments and radiant heat by activating IL-10 and β-endorphin, which was blocked by IL-10 and β-endorphin antibodies (Tang et al., 2020). Therefore, Mor exerts therapeutic effects in neuropathy via the spinal microglial expression of IL-10 and β-endorphin after GLP-1 activation.

#### 3.1.4 Other neuroprotective effects of Mor

In H_2_O_2_-induced SH-SY5Y cells (a subline of human neuroblastoma cells), Mor increased GSH, SOD, and mitochondrial membrane potential and reduced reactive oxygen species (ROS), nitrite content, lipid peroxidation, and intracellular accumulation of Ca^2+^. Moreover, Mor upregulated Bcl-2 and decreased caspase-3, caspase-9, and Bax (Wang et al., 2008; Wang et al., 2009; Zhang et al., 2017). In rat pheochromocytoma cells (PC12 cells), which are used to study neurotoxic activity (Wiatrak et al., 2020), Mor upregulated Bcl-2, downregulated Bax, cytochrome C, and cleaved caspase-3, alleviated cell death, and decreased the phosphorylation of JNK and p38MAPK simultaneously (Chen et al., 2018). The results demonstrated that Mor inhibits the activation of apoptosis, inflammatory, and oxidative stress to protect nerve cells.

In depression model rats established by forcibly disrupting their regular routine, Mor improved the behavioral test score in open-field and sugar water preference experiments, reduced pathological damage to the prefrontal cortex, and decreased levels of IL-1β, TNF-α, and NF-κBp65, implying that Mor exerts neuroprotective effects by reducing neuroinflammatory (Mo, 2020).

Mor demonstrates the ability to protect neurons and decelerate the progression of Parkinson’s disease (PD). In the 1-methyl-4-phenyl-1,2,3,6-tetrahydropyridine (MPTP)-induced PD model, Mor activates the Nrf2/HO-1 signaling pathway, leading to the decrease of MDA, ROS production, lipid peroxidation, and mitochondrial damage and the increase of GSH and GPX4, to prevent ferroptosis caused by lipid oxidation (Li M. et al., 2023).

### 3.2 Effects of Mor on bone protection

**FIGURE 4:**
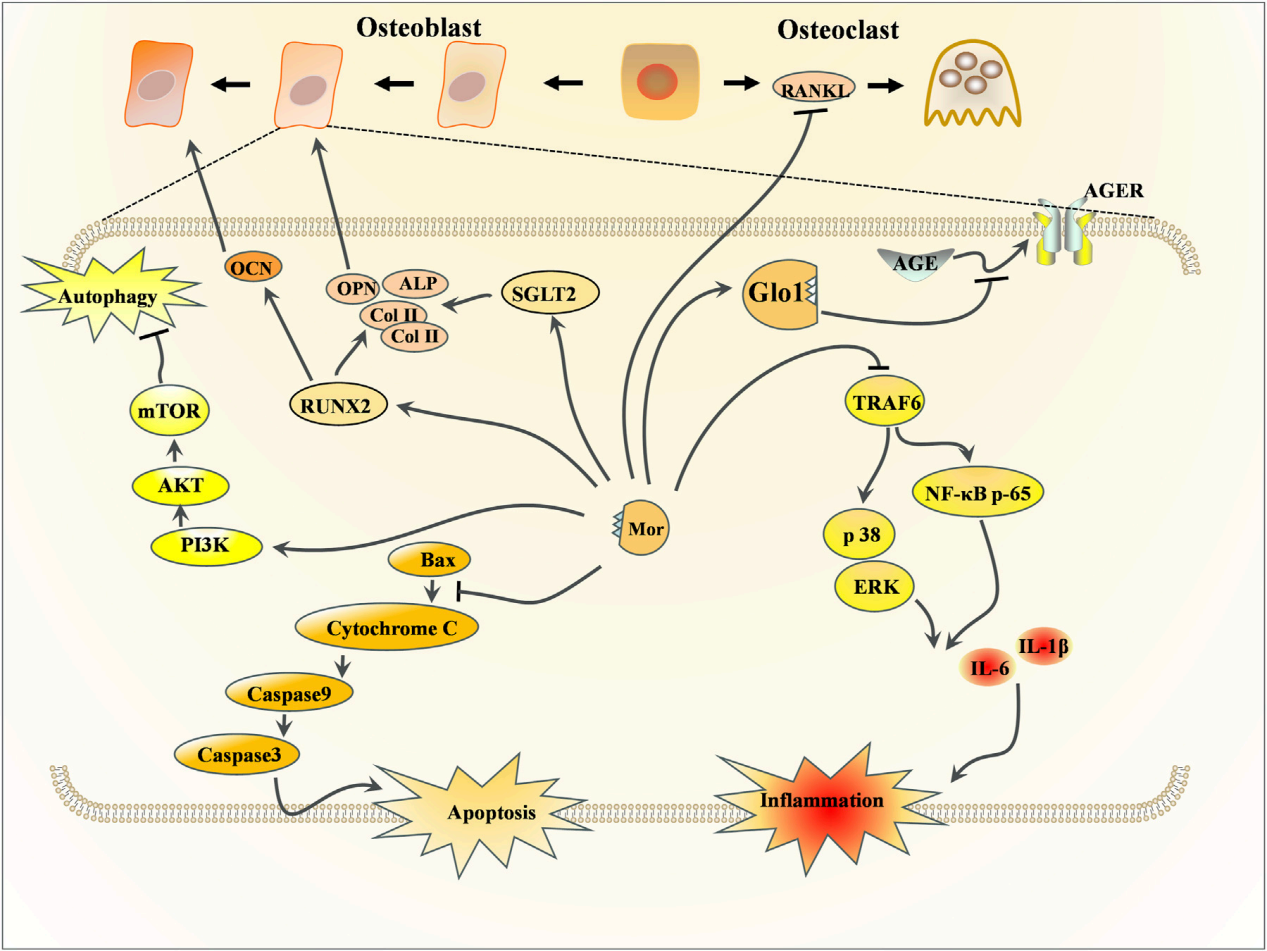
Effects of Mor on osteoporosis.

**FIGURE 5:**
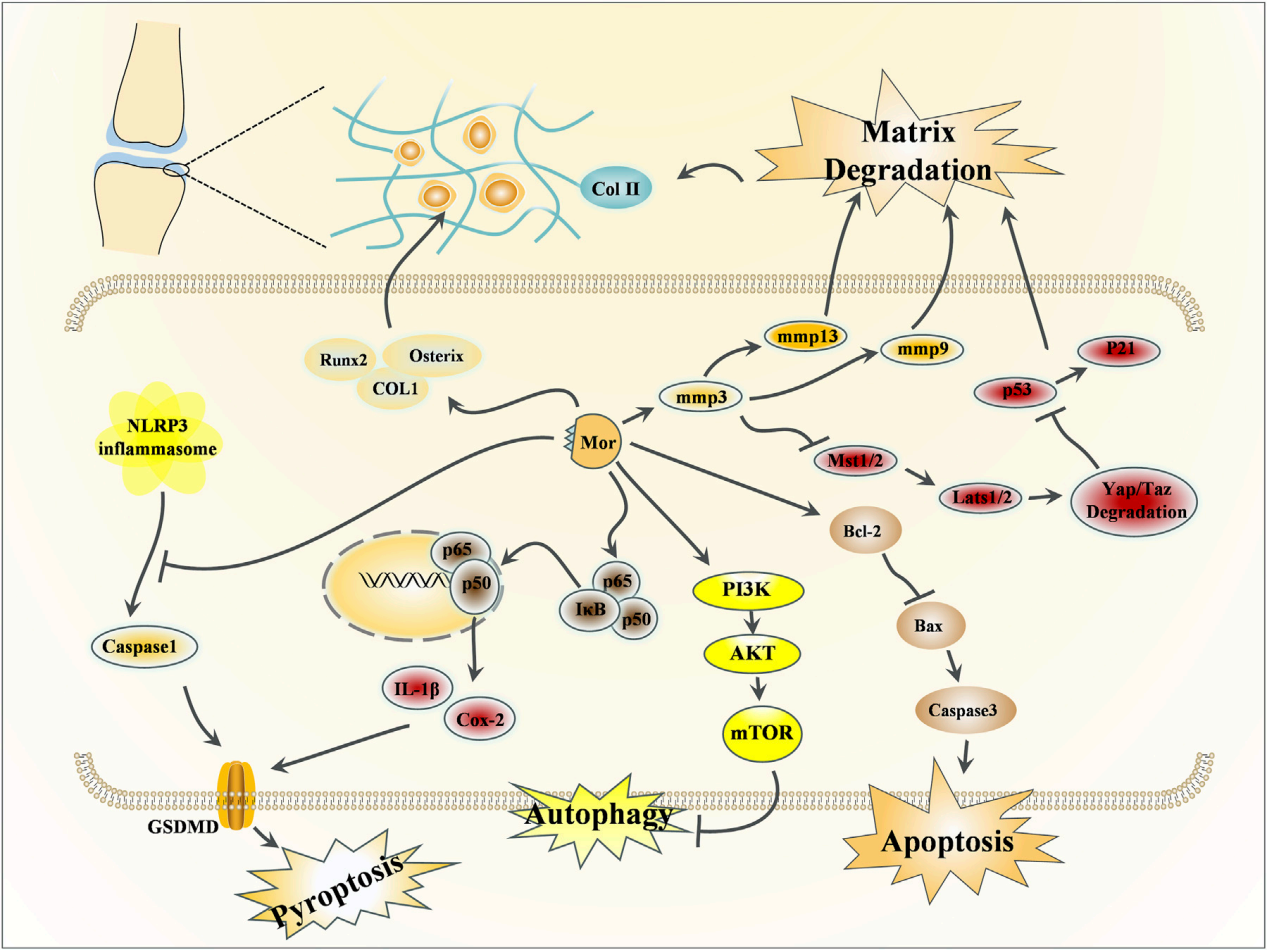
Effects of Mor on osteoarthritis.

In TCM, the kidney is believed to influence bone, and CF’s function in nourishing the kidney is believed to have a miraculous effect on strengthening bones. Bone-related diseases can be classified into osteoporosis and osteoarthritis; the former is related to bone metabolism (Lane, 2006), while the latter is primarily associated with cartilage (Glyn-Jones et al., 2015). This section discusses the protective effects of Mor in osteoporosis and osteoarthritis, the detailed pharmacological effects was listed in Table 4, and the detailed mechanism of action can be seen in Figures 4, 5.

**TABLE 4:** Bone protection.

Cell or animal model and method	Application	Dose	Molecular mechanism	Reference
MC3T3-E1 cell	*In vitro*	1, 10, and 100 μg/mL	ALP↑, Col-I↑, osteocalcin↑ caspase-3↓, caspase-9↓, RANKL↓; Bcl-2↑	Li et al. (2010)
MC3T3-E1 cell	*In vitro*	1, 20, and 100 µM	Osteoblastic differentiation: ALP↑ OCN↑, Runx2↑, Col-I↑, osterix↑ p-PI3K↑, p-Akt↑, p-mTOR↑, p-p70S6K↑	Liu et al. (2021a)
OVX-induced mice	*In vivo*	60 μg/kg	OCN↑ PI3K↑, mTOR↑	Liu et al. (2021a)
MC3T3-E1 cell primary osteoblasts	*In vitro*	2, 10, and 20 µM	Osteoblastogenesis: Alpl↑, Runx2↑, and Sp7↑ osteoclast: Nfatc1↓, Ctsk↓, MMP9↓, and Acp5↓ osteoblastic differentiation: ALP↑, TRAP↓	Lee et al. (2021)
OVX-induced mice	*In vivo*	2 and 10 mg/kg	Bone mineral density↑, bone structural compartment ↑	Lee et al. (2021)
HG induced BMSC	*In vitro*	1, 10, and 100 µM	Osteogenic: ALP↑, Bmp2↑, Col-I↑, Opn↑, Ocn↑, Runx2↑ AGEs↓, RAGE↓	Sun et al. (2020)
A rat type 1 diabetic mice	*In vivo*	15 and 30 μg/kg	Col-I↑, GLO1↑ and RAGE↓	Sun et al. (2020)
Chondrocytes cell	*In vivo*	0.1, 20, and 100 μM	PCNA↑, type II collagen↑, aggrecan↑ AKT↑, ERK↑	Cheng et al. (2015)
DMM-induced rats	*In vivo*	0.01, 0.5, and 10 mg/kg	p-AKT↑, p-ERK↑	Cheng et al. (2015)
IL-1β-induced chondrocytes Mor	*In vitro*	2, 10, and 50 µM	Cox-2↓, MMP-3↓, MMP-13↓, PGE2↓, collagenase↓	Park et al. (2021b)
DMM-induced rats	*In vivo*	5 and 20 mg/kg	Cox-2↓, MMP-3↓, MMP-13↓	Park et al. (2021b)
DMM-induced mice	*In vivo*	4 and 20 μg/kg	Col-II↑, MMP13↑; p-I-κBα↓, p65↓	Yu et al. (2021)
IL-1β-stimulated chondrocytes	*In vitro*	20,100 μg/mL	Col-II↑, MMP13↑; cleaved caspase-3↓; cleaved caspase-1↓, NLRP3↓, GSDMD↓	Yu et al. (2021)
Chondrocytes cell	*In vitro*	1, 20, and 100 µM	Autophagic: p-PI3K↑, p-AKT↑, mTOR↑	Xiao et al. (2020)

#### 3.2.1 Effects of Mor on osteoporosis

Osteoporosis typically occurs in elderly and menopausal women, whereas idiopathic osteoporosis primarily occurs in adolescents. *CF* and Liu Wei Di Huang Wan are commonly used to treat postmenopausal osteoporosis (Gong et al., 2019; Kim et al., 2022).

Mouse embryo osteoblast precursor cells (MC3T3-E1 cells) can differentiate into osteoblasts and osteocytes, and they co-regulate bone formation and dissolution (Xu et al., 2020). Mor itself exerts no effects on the proliferation of MC3T3-E1 cells (Li et al., 2010); however, among 14 Mor derivatives, the introduction of a methyl group at position 7 and substitution with the beta configuration proved to be better than the alpha configuration (Han et al., 2018).

Mor can promote bone formation by increasing bone formation markers (ALP, Col-I, and osteocalcin) in MC3T3-E1 cells. Mor also upregulates Bcl-2 and downregulates caspase-3, caspase-9, and RANKL (Li et al., 2010). PI3K and mTOR inhibitors reversed MC3T3-E1 cell differentiation to mature osteoblasts, and mTOR overexpression can enhance osteoblast differentiation suppressed by PI3K inhibitors (Liu et al., 2021a). Hence, the anti-osteoporosis mechanism of Mor may be associated with apoptosis and autophagy. Additionally, osteoblast formation enhanced by Mor was also reversed by PI3K or mTOR inhibitors (Liu et al., 2021a). Further studies have found that Mor can promote mTOR activity and autophagy; Beclin1 or Atg13 agents, which enhance autophagy, may help improve protein levels in MC3T3-E1 cells. Moreover, Mor enhanced Atg13 expression, and mTOR overexpression reversed the expression of Beclin1. TAT-Beclin1 (inducers of autophagy) amplified the positive effect of Mor on bone parameters, such as the trabecular area and OCN expression in OVX mice (Li et al., 2022).

In ovariectomy-induced mice, the micro-CT images indicated that Mor reversed bone mineral density decline and structural compartment loss (Lee et al., 2021).

Mor shortened the adherence time of bone marrow stromal cells (BMSCs) and accelerated the formation of typical BMSC colonies to promote bone formation (Hu et al., 2013). High glucose causes the dysfunction of bone formation in type 1 diabetes mellitus. In high glucose-induced BMSCs, Mor reversed the osteogenic differentiation of BMSCs and increased the expression of osteospecific genes (*Alp*, *Bmp2*, *Col-I*, *Opn*, *Ocn*, and *Runx2*). In addition, Mor suppressed advanced glycation end product (AGE) formation and receptor for advanced glycation end product (RAGE) expression by triggering Glo1, whereas the Glo1 inhibitor (BBGCP2) partially reversed the suppressive effect of Mor on AGE-RAGE signaling (Uribarri et al., 2007).

In the type 1 diabetic rat model, Mor improved the mass and microarchitecture of the distal femur trabecular bone, as well as the bone volume/total volume, trabecular number, trabecular thickness, cortical bone area, and cortical thickness in the micro-CT image. Moreover, AGE-RAGE, Glo1, and osteo-related proteins, including RUNX2, Glo1, and Ocn in rat distal femurs, were all regulated by Mor. Therefore, Mor attenuated HG-mediated BMSC dysfunction partly by inhibiting AGE-RAGE signaling and activating Glo1 (Sun et al., 2020).

Mor attenuated chronic inflammation-triggered bone loss in mouse models by enhancing bone density and bone microstructure and inhibiting the expression of IL6, IL1β, and ALP. Moreover, in BMSCs, Mor downregulated IL-6 and IL-1β and upregulated the osteogenic mediators Runx2 and OCN. These effects were attributed to its inhibition of TRAF6-mediated NF-κB and MAPK signaling pathways (Xiao et al., 2023).

In the glucocorticoid-induced osteoporosis zebrafish model, MOR improved vertebral loss and increased the expression of osteoblastogenesis factors such as ALP, Runx2, and Col-I. Additionally, sodium-glucose cotransporter 2 (SGLT2) played a vital role in the anti-osteoporosis of Mor (Yang et al., 2023).

#### 3.2.2 Effects of Mor on osteoarthritis

Osteoarthritis affects the elderly population, physical activity, and metabolic syndrome. The leading causes of osteoarthritis are degenerative lesions and persistently low levels of joint inflammation and matrix degradation (Abramoff and Caldera, 2020).

Chondrocytes are directly responsible for cartilage formation, metabolism, and repair (Chen H. et al., 2021). Mor could enhance chondrocyte viability and promote matrix synthesis by upregulating the expression of PCNA (responsible for proliferative activity), Col-II (the foundation of cartilage), and aggrecan (the key proteoglycan in articular cartilage). Furthermore, Mor activated Akt and Erk (Cheng et al., 2015).

In IL-1β-induced chondrocytes, Mor inhibited inflammation by downregulating Cox-2, MMP3, and MMP13. PGE2 and collagenase were reduced following treatment with Mor (Park E. et al., 2021). In another study, Mor inhibited apoptosis by reducing cleaved caspase-3 expression and suppressed pyroptosis by decreasing cleaved caspase-1, NLRP3, and GSDMD expression; in addition, Mor stimulated cartilage matrix synthesis by regulating Col-II and MMP13 (Yu et al., 2021). Studies have verified the protective effects of Mor by inhibiting autophagy in chondrocytes via the PI3K/mTOR pathway. PI3K and mTOR inhibitors significantly reversed the autophagy suppressed by Mor but did not affect the protective role of Mor in chondrocytes (Xiao et al., 2020).

Medial meniscus (DMM)-damaged mice are established by opening the medial joint capsule of the knee and transecting the medial meniscus after opening the medial meniscotibial ligament (Glasson et al., 2007). In this model, intra-articular injection of Mor elevated the level of proteoglycans in the cartilage matrix and ameliorated the cartilage damage. In addition, Mor increased Akt and Erk expression (Cheng et al., 2015) and inhibited sclerosis and cartilage degradation by reducing inflammatory mediators, such as Cox-2, MMP3, and MMP13 (Park E. et al., 2021).

Micro-CT imaging has shown that Mor attenuated osteoarthritis progression and stimulated cartilage matrix synthesis by increasing Col-II and decreasing MMP13. Mor also reduced p-IκBα and reversed the translocation of p65 into the nucleus; Mor might inhibit chondrocyte pyroptosis and apoptosis by inhibiting NF-κB signaling from preventing cartilage matrix degradation (Yu et al., 2021).

The prolonged use of glucocorticoids induces osteonecrosis of the femoral head (GIONFH). In dexamethasone-induced dysfunction in stem and endothelial cells and GIONFH rats, Mor mitigated stem and endothelial cell dysfunction through the PI3K/AKT and Bax/Bcl-2/Caspase3 signaling pathway, suggesting that Mor may be a potential agent for GIONFH (Jiang et al., 2024).

Intervertebral disc (IVD) degeneration (IVDD) is a leading cause of chronic low back pain and disability. Nucleus pulposus (NP) cell senescence is closely related to IVDD. In lumbar spine instability surgery-induced mice and H_2_O_2_-induced NP cells, Mor has been found to reduce SA-β-gal activities and the expression of p53 and p21, which are indicators of senescence. Mor suppressed the activation of Hippo signaling by inhibiting p-Mst1/2 and p-Lats1/2 and increasing Yap/Taz. In the mouse IVDD model, the inhibition of Hippo signaling by Mor was further confirmed. Mor protected against NP cell senescence to alleviate IVDD progression by inhibiting the ROS-Hippo-p53 pathway (Zhou et al., 2022).

### 3.3 Renoprotective effects of Mor

**FIGURE 6:**
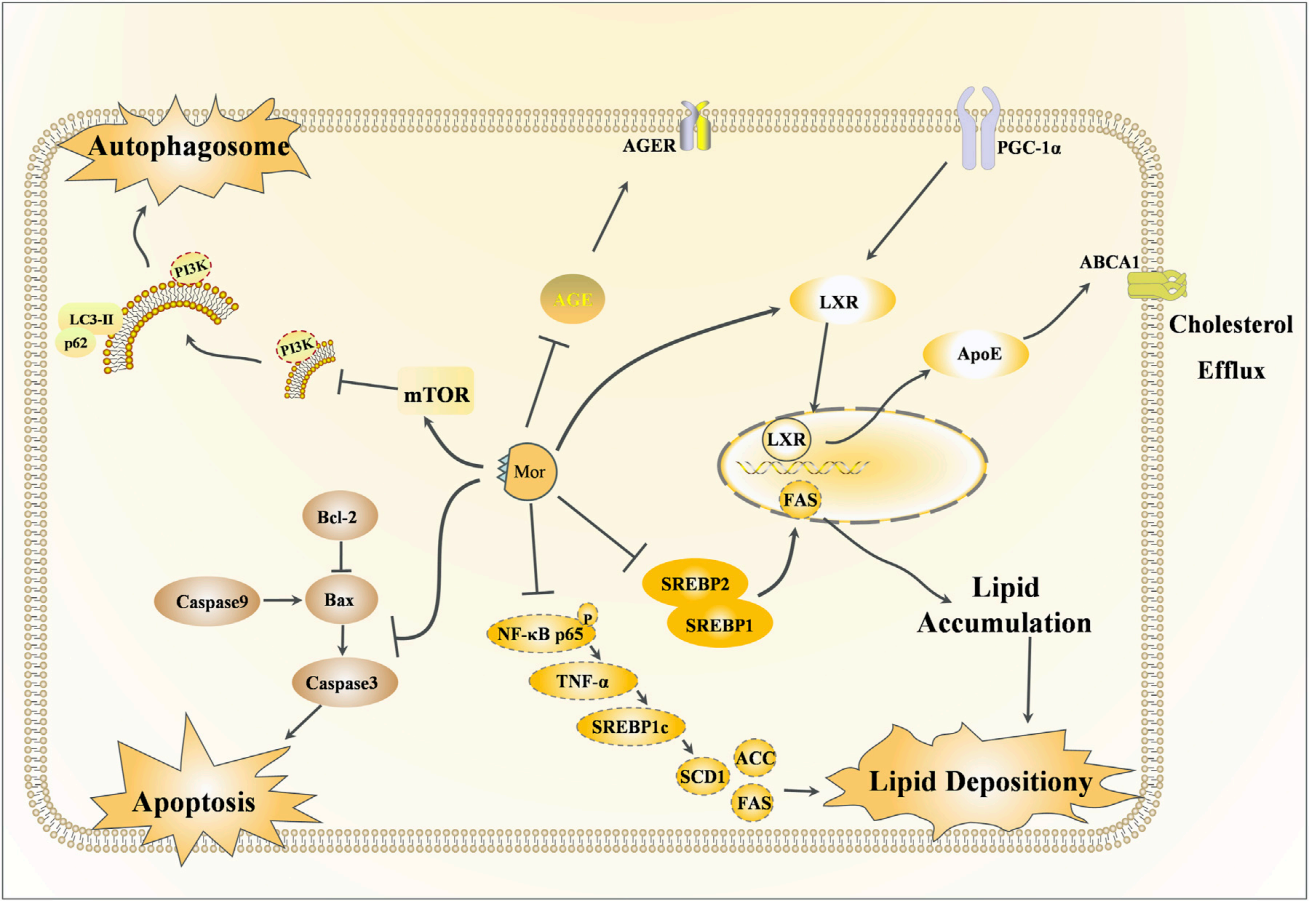
Renoprotective effects of Mor.

Along with the main indications of *CF*, Mor also has a therapeutic effect on diabetes, which is referred to as Xiao-Ke in TCM. The detailed mechanism of action of the rephroprotective action is shown in Figure 6 with the detailed pharmacological effects was listed in Table 5. With the intervention of Mor, human umbilical vein endothelial cell (HUVEC) survival was recovered from high ambient glucose, the number of cells in the S-phase was increased, and morphological damage was alleviated, suggesting that Mor can inhibit diabetic angiopathies (Xu et al., 2004). In the AGE-induced rat renal mesangial cell model, Mor improved cell morphology, inhibited proliferation, and reduced oxidative stress by regulating ROS, SOD, and GSH (Xu et al., 2006). Moreover, Mor recovered the morphological damage and cellular ultrastructure in AGE-induced rat glomerular mesangial cells and inhibited RAGE, p38MAPK, NF-κB, and TGF-β expression (Lv et al., 2016).

**TABLE 5:** Kidney protection.

Cell or animal model and method	Application	Dose	Molecular mechanism	Reference
HUVEC was incubated in glucose	*In vitro*	1, 10, and 100 µM	Rate of cells into S-phase↓, morphological damage↓	Xu et al. (2004)
AGE-induced GMCs	*In vitro*	0.1, 1, and 10 µM	RAGE↓, p38MAPK↓, NF-κB↓, TGF-b↓	Lv et al. (2016)
db/db mice	*In vivo*	20 and 100 mg/kg	SREBP-1 ↓SREBP-2↓ NF-κB↓, Cox-2↓, iNOS↓	Park et al. (2010)
H_2_O_2_-injured podocytes	*In vitro*	2.5 and 5 µM	Apoptosis: Bax↓, Bax/Bcl-2↓ caspase-3↓, cleaved-caspase-3↓, NOX4↓ Autophagy: p62↓, LC3-II↑, mTOR↓, p-mTOR↓	Gao et al. (2020)
PA- or HG-induced mRTECs	*In vitro*	0.5 and 1 µM	Lipid metabolism: PGC-1α↑, LXR↑, ABCA1↑, ABCG1↑, ApoE↑	Gao et al. (2021)
KKAy mice	*In vivo*	50 mg/kg	Lipid metabolism: blood glucose↓, Scr↓, UACR↓, TC↓, TG↓ P–NF-κBp65↓, TNF-α↓, SREBP1c↓, ACC↓	Zhu et al. (2023)
PA- or HG-induced podocyte cells	*In vitro*	1, 2, and 4 µM	P–NF-κBp65↓, TNF-α↓, SREBP1c↓, ACC↓	Zhu et al. (2023)
KKAy mice	*In vivo*	50 mg/kg	Lipid dysregulation: blood glucose↓, TC↓, TG↓, LDL-C↓, Scr↓, UACR↓ accumulation of glycogen: PGC-1α↑, PPARγ↑, CD36↓, LXRs↑, ABCA1↑ and nephrin↑	Chen et al. (2024b)
PA-stimulated HK-2 cells	*In vitro*	0.5, 1, and 2 µM	Accumulation of glycogen:PGC-1α↑, PPARγ↑, CD36↓, LXRs↑, ABCA1↑ and nephrin↑	Chen et al. (2024b)

In streptozotocin-induced diabetes mellitus rats, Mor inhibited hyperglycemia by decreasing serum glucose and urinary protein, increasing serum albumin and total protein, and reducing serum urea nitrogen, creatinine clearance, serum glycosylated protein, and serum and renal thiobarbituric acid reactive substances. Moreover, the expressions of AGEs and AGER decreased in diabetic rats after Mor administration (Yokozawa et al., 2008).

Db/db mice have gene-encoding mutations at the leptin receptor, with high susceptibility to obesity and type 2 diabetes mellitus, and hence are suitable for the study of type 2 diabetes mellitus and metabolic liver and kidney disease (Suriano et al., 2021). In db/db mice, Mor decreased the overproduced glucose, triglyceride, and cholesterol and the expression of SREBP-1, SREBP-2, ROS, and TBARS (a by-product of lipid peroxidation) and increased GSH/GSSG. Moreover, Mor reduced NF-κB, Cox-2, and iNOS. Therefore, Mor inhibits metabolic disorders (hyperglycemia and dyslipidemia), oxidative stress, and inflammation in diabetic kidneys (Park et al., 2010; Yokozawa et al., 2010).

Podocyte, a type of glomerular visceral epithelial cell, play a critical role in filtration and are involved in glomerulopathies induced by diabetic nephropathy; in H_2_O_2_-injured podocytes, Mor reduced apoptosis by downregulating Bax and decreasing the Bax/Bcl-2 ratio. Furthermore, Mor inhibited autophagy by increasing LC3-II expression and reducing p62, mTOR, and NOX4. Additionally, Mor restrained the apoptosis by suppressing caspase-3 and cleaved caspase-3 (Gao et al., 2020).

In mouse renal tubular epithelial cells (mRTECs) damaged by sodium palmitate or HG, Mor reduced the accumulation of lipids and cholesterol by upregulating PGC-1α, LXR, ABCA1, ABCG1, and ApoE. This suggests that Mor promotes cholesterol efflux in mRTECs via the PGC-1α/LXR pathway (Gao et al., 2021).

In KKAy mice, which is an ideal animal model for early-to-mid-stage type 2 diabetic nephropathy, Mor normalized renal lipid metabolism by improving podocyte cholesterol efflux and regulating podocyte cholesterol uptake through upregulating the PGC-1α/LXRs/ABCA1 and PGC-1α/PPARγ/CD36 signaling pathways, respectively. In podocyte cells induced with PA or HG, Mor alleviated lipid accumulation through the exact mechanism (Yu et al., 2024).

Mor reduces lipid deposition in diabetic nephropathy by inhibiting the NF-κB/TNF-α/SREBP1c signaling pathway. In KKAy mice and PA-stimulated HK-2 cells, Mor inhibited the activation of NF-κBp65, reduced the levels of TNF-α and SREBP1c, lowered the production of lipid components such as ACC, FAS, and SCD1, and ultimately reduced renal lipid accumulation (Zhu et al., 2023).

### 3.4 Hepatoprotective effects of Mor

**FIGURE 7:**
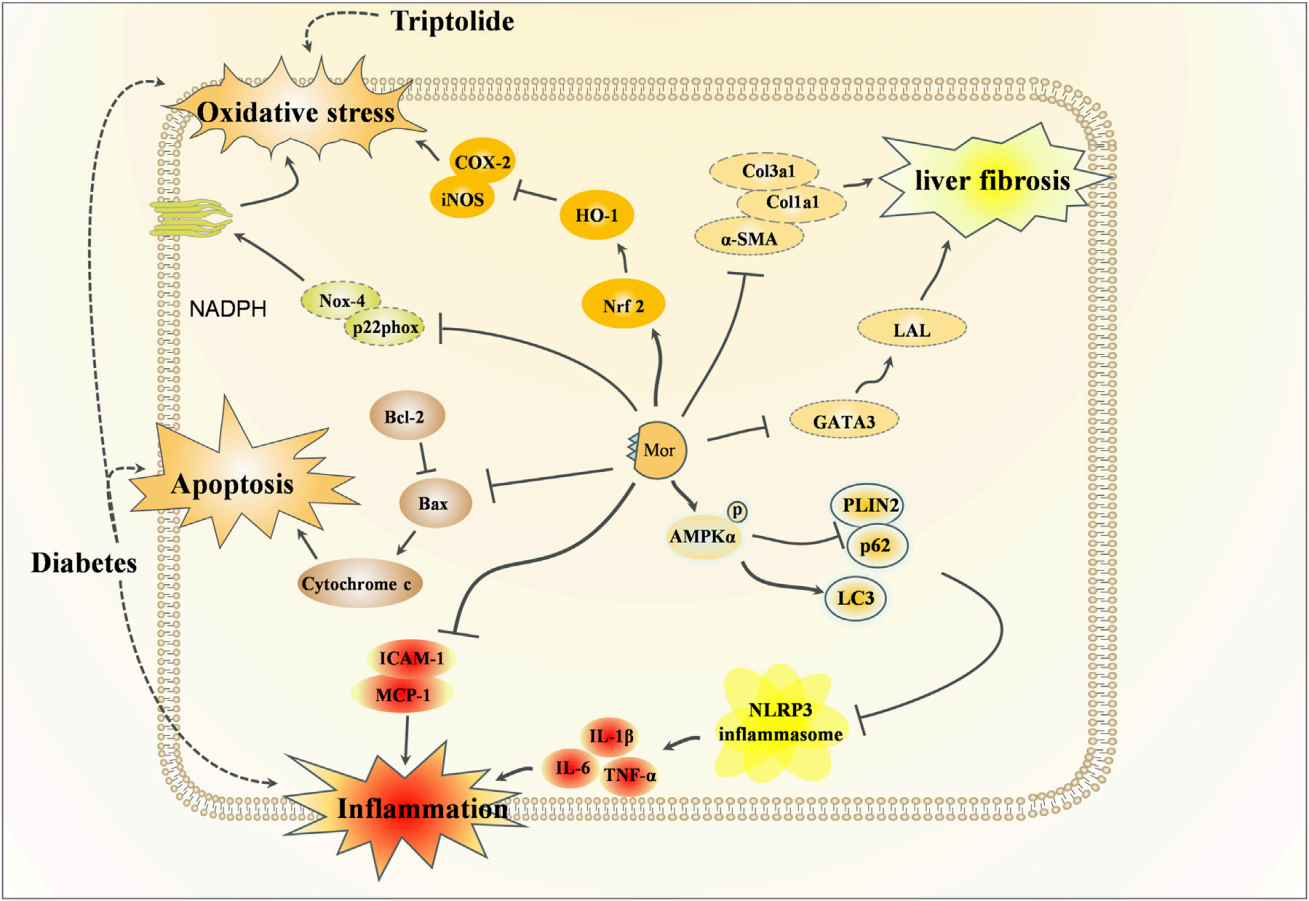
Hepatoprotective effects of Mor.

In traditional Chinese medicine theory, CF is considered a liver-nourishing tonic. Modern clinical trials have also confirmed its hepatoprotective effect (Sangsefidi et al., 2021; Bayram et al., 2024). There are also an increasing number of studies on the hepatoprotective effects of Mor, and the detailed mechanism of hepatoprotective action is shown in Figure 7, with the detailed pharmacological effects was listed in Table 6.

**TABLE 6:** Hepatoprotective effects of Mor.

Cell or animal model and method	Application	Dose	Molecular mechanism	Reference
db/db mice	*In vivo*	20 and 100 mg/kg	Oxidative stress: Nox-4↓, p22phox↓, Nrf2↓, HO-1↓, NF-kB↓, Cox-2↓, iNOS↓ Inflammation: MCP-1↓, ICAM-1↓ Apoptosis: Bax↓, cytochrome c↓	Park et al. (2011)
Triptolide-injured mice	*In vivo*	5 mg/kg	ALT↓, AST↓	Zhou et al. (2018b)
Triptolide-injured HepG2 cells	*In vitro*	5 µM	Oxidative stress: Nrf2↓, HO-1↓	Zhou et al. (2018b)
TGF-β1-induced HSC-T6 cell	*In vitro*	5, 10, and 20 µM	HSC activation:α-SMA↓, Col1a1↓, Col3a1↓, Lipa↓ Lipid metabolism: GATA3↓, LAL↓	An et al. (2022)
HFFD-driven NASH in mice	*In vivo*	10 and 20 mg/kg	AST↓, ALT↓, TG↓, T-CHO↓, LDL-c↑, HDL-c↓; p-AMPKα↑ Hepatic lipophagy and fatty acid oxidation: PLIN2↓, ATG12-ATG5↑, p62↓, LC3↑, PPARα↑ and CPT1α↑ Oxidative stress and inflammatory: NLRP3↓, ASC↓, Caspase-1 p20↓, and IL-1β↓	Zhang et al. (2024)
PA-induced HepG2 cells	*in vitro*	25 and 50 µM	Hepatic lipophagy and fatty acid oxidation: PLIN2↓, p62↓, LC3↑, PPARα↑ and CPT1α↑ Oxidative stress and inflammatory: NLRP3↓, ASC↓, Caspase-1 p20↓, and IL-1β↓	Zhang et al. (2024)

Diabetes damages the structure and function of the liver, and in turn, liver injury impairs glucose tolerance and causes diabetes. Using db/db mice, Mor has been shown to reduce the levels of glucose, triglyceride, and total cholesterol; regulate the activity of ROS, GSH, and GSSG (Park et al., 2009); and inhibit the expression of NF-κB, Cox-2, iNOS, and SREBP-1 and -2 (promotors of the expression of genes related to fatty acid synthase and cholesterol (Brown and Goldstein, 1997) but increase the expression of PPARa, which is related to the anti-obesity and the regulatory enzymes of fatty acid oxidation (Lin et al., 2022). Mor decreased the levels of glucose, ALT, and AST in serum, as well as ROS and lipid peroxidation in the liver of db/db mice, and significantly reduced the expression of oxidative stress-related proteins, such as NADPH oxidase subunits (Nox-4 and p22phox), Nrf2, HO-1, NF-κB, Cox-2, and iNOS, inflammation-related proteins (MCP-1 and ICAM-1) and apoptosis-related proteins (Bax and cytochrome C) (Park et al., 2011). Mor can regulate lipid metabolism, inflammation, and oxidative stress in the liver to improve diabetes.

In triptolide-induced liver injury, Mor reduced the levels of ALT and AST. *In vitro*, Mor reversed the growth inhibition, morphological changes, apoptosis, and nucleus deformation of HepG2 cells it also restored the expression of Nrf2 and HO-1, indicating that Mor improved triptolide-induced liver injury by preventing or alleviating oxidative stress (Zhou Y. et al., 2018).

In CCl4 and HFD-induced liver fibrosis mice, Mor reduced α-SMA, collagen, and GATA expressions by targeting GATA3 and LAL, thereby inhibiting HSC activation (An et al., 2022). In the NASH mouse model established by a high-fat and high-fructose diet, Mor attenuated hepatic lipid metabolism disorders and inhibited NLRP3 inflammasome activation by promoting AMPKα phosphorylation-mediated lipophagy and fatty acid oxidation. The consistent results performed in PA-treated cell models suggest that Mor may be a potential agent to inhibit NASH progression by promoting lipophagy and inhibiting inflammasome activation (Zhang et al., 2024).

Insulin resistance is another factor involved in diabetes. In HepG2 cells, Mor promoted glucose uptake, as well as in the HepG2 cell insulin resistance model. The results indicate that Mor can lower blood sugar by improving insulin resistance to alleviate type 2 diabetes-associated symptoms (Huang et al., 2017).

### 3.5 Cardiovascular protective effects of Mor

Acute myocardial infarction remains a major cause of mortalityworldwide despite substantial improvements in prognosis (Reed et al., 2017). The specific pharmacological effects of MOR in cardiovascular protective function are shown in Table 7. In STZ combined with high-fat diet-induced T2DM mice, Mor reduced TC and increased SOD and NOS (Feng et al., 2016), prevented HG-induced rat cardiomyocyte injury, and reduced apoptosis by modulating Bax and Bcl-2 and decreasing caspase-3 (Pi et al., 2017). In STZ combined with MACO mice, Mor improved heart function, reduced Mb, CK-MB, and cTNⅠ, regulated inflammation and oxidative stress by reducing IL-6, TNF-α, and MDA and enhancing IL-10 and SOD, and increased the expression of AMPKα1, PGC-1α, and GLUT4 (Li et al., 2018).

**TABLE 7:** Cardiovascular protective effects of Mor.

Cell or animal model and method	Application	Dose	Molecular mechanism	Reference
STZ combined with high-fat diet-induced T2DM C57BL/6J mice	*In vivo*	20 and 80 mg/kg	Body weight↑, blood glucose↑, heart-to-body ratio↑ TC↓, SOD↑, and NOS↑	Feng et al. (2016)
HG-induced rat cardiomyocyte injury	*In vitro*	25, 50, and 100 μg/mL	Cell viability↑, apoptosis cell↓ ROS↓, Bax/Bcl-2↓ and caspase-3↓	Pi et al. (2017)
STZ combined with MACO mice	*In vivo*	100 mg/kg	Mb↓, CK-MB↓, and cTNⅠ↓ Inflammatory: IL-6↓, TNF-α↓ and IL-10↓ Oxidative stress: MDA↓ and SOD↑ AMPKα1↑, PGC-1α↑ and GLUT4↑	Li et al. (2018)
AMI SD rats	*In vivo*	45, 90, and 180 mg/kg	Oxidative stress: SOD↑, MDA↓ Apoptosis: Bcl⁃2↑, Bax↓, cleaved caspase⁃3↓, and cleaved-caspase⁃8↓	Cui et al. (2018)
AMI SD rats	*In vivo*	45, 90, and 180 mg/kg	CK-MB↓, LDH↓, ɑ- HBDH↓, and AST↓ NF-κB↓	Yu and Wang (2018)
AMI SD rats	*In vivo*	45, 90, and 180 mg/kg	LDH↓ and cTnT↓ IL-6↓, IL-1β↓ and TNF-α↓	Yu et al. (2018)
AMI SD rats	*In vivo*	45, 90, and 180 mg/kg	Apoptosis: Bcl⁃2↑, Bax↓, caspase⁃3↓, and caspase⁃9↓ Cardiomyocyte proliferation: Ki67 ↑ and PCNA↑ Myocardial damage: CK⁃MB↓, cTnΙ↓ and Mb↓ Inflammatory: TNF⁃α↓, IL⁃6↓, IL⁃4↑, and IL-10↑ Oxidative stress: SOD↑, GSH↑ and MDA↓, Nrf2↑, HO -1↑, and NQO1↑	Zhou et al. (2019)
AMI SD rats	*In vivo*	30, 60, and 120 mg/kg	Inflammatory: IL-8↓, IL-17↓ and hs-CRP↓ Bcl-2 ↑, Bax ↓, cleaved caspase-3 ↓ Smad3 ↓Smad2 ↓ and TGF-β1 ↓	Chen et al. (2021b)
Rat cardiomyocyte line H9c2 deprived of oxygen and glucose	*In vitro*	100 μg/mL	Autophagy: LC3II↑, LC3I↑, and cleaved-PARP↑ Apoptosis: p-JNK↓, p-BCL2-S70↑ and p-BCL2-S87↑, caspase-3 ↓	Ke et al. (2023)
AMI SD rats	*In vivo*	60, 120, and 240 mg/kg	Cell cycle activity of NRCMs↑ cardiomyocyte mitosis↑ cyclin D1↑, CDK4↑, cyclin A2↑ and cyclin B1↑	Zheng et al. (2023)
Neonatal rat primary cardiomyocytes (NRCMs) deprived of oxygen and glucose	*In vitro*	1, 10, and 100 µM	Cell cycle activity of NRCMs↑ cyclin D1↑, CDK4↑, cyclin A2↑ and cyclin B1↑	
AMI SD rats	*In vivo*	45, 90, and 180 mg/kg	Ang-1↑ and FGF-2↑	Wang et al. (2019)
AMI SD rats	*In vivo*	45, 90, and 180 mg/kg	Newly generated endothelial cells ↑ VEGFA↑, p-VEGFR2↑, p-PKC↑, p-Src↑ and p-Erk1/2↑	Liu et al. (2020)
Rat coronary artery endothelial cells deprived of oxygen and glucose	*In vitro*	1, 10, and 100 µM	VEGFA↑, p-VEGFR2↑, and p-Erk1/2↑	

Morroniside can prevent myocardial injury due to ischemia and hypoxia. In an acute myocardial infarction (AMI) rat model induced by ligating the anterior descending coronary artery, Mor exhibited cardioprotective effects by improving cardiac function and decreasing CK-MB, LDH, ɑ-HBDH, AST, and cTnT; this protection may be related to anti-inflammatory activity. Mor decreased IL-6, IL-1β, and TNF-α, NF-κB (Yu and Wang, 2018; Yu et al., 2018) but increased IL-4 and IL-10 (Zhou et al., 2019). Moreover, Mor reduced apoptosis by regulating Bax and Bcl-2 (Bai et al., 2017) and caspase-3, caspase-8, and caspase-9 (Cui et al., 2018; Zhou et al., 2019) and alleviated oxidative stress by increasing SOD, GSH, Nrf2, and HO-1 while lowering MDA (Zhou et al., 2019). Another study found that Mor relieved inflammatory factors such as IL-8, IL-17, and hs-CRP and attenuated myocardial fibrosis by the downregulation of Smad3, Smad2, and TGF-β1 (Chen Z. et al., 2021). In H9c2 cells, Mor can inhibit cardiomyocyte apoptosis and autophagic death caused by hypoxia by inhibiting p-JNK and p-BCL2 at Ser70 and Ser87 and dissociation of BCL2–Beclin1 and BCL2–Bax complexes. In addition, Mor ameliorated the reduction of mitochondrial membrane potential (Ke et al., 2023). Morroniside could enhance cardiomyocyte cell cycle activity by upgrading cell cycle proteins (cyclin D1, CDK4, cyclin A2, and cyclin B1) in AMI rats (Zheng et al., 2023).

In the infarct border zone of AMI rats, Mor improved the level of Ang-1 and FGF-2, indicating that Mor promoted angiogenesis (Wang et al., 2019). Mor could also increase the newly generated endothelial cells and arterioles simulated by vascular endothelial growth factor A (VEGFA), an inducer and regulator of angiogenesis, and vascular endothelial growth factor receptor 2 (VEGFR2). The results were verified in rat coronary artery endothelial cells deprived of oxygen and glucose (Liu et al., 2020).

In adenosine diphosphate-induced rabbits, Mor has anti-coagulant properties in adenosine diphosphate, arachidonic acid, and platelet, activating factors that induce platelet aggregation (Wang X. et al., 2010). Mor also inhibited the increase in platelet Ca^2+^ (Ai et al., 2012) and decreased cyclooxygenase (Sun et al., 2012) and thromboxane B2 (Zuo et al., 2012) levels. In MACO rats, Mor reduced platelet aggregation (Cheng et al., 2013), whole blood and plasma viscosity (Pan et al., 2013), fibrinogen and prothrombin time and activated partial thromboplastin time, thrombin time (Yuan et al., 2013), and hematocrit percentage (Ai et al., 2014). These results suggest that Mor can improve cardiovascular and cerebrovascular diseases by altering the function of blood clotting.

### 3.6 Protective effect of Mor on the digestive system

In rats with chronic atrophic gastritis, Mor increased levels of gastrin and decreased levels of motilin. Mor also reduced inflammation by lowering TNF-α, IL-6, and IL-1β. In gastric mucosal cells, Mor decreased the expression of Bax, cleaved caspase-3, cleaved caspase-9, p-NF-κBp65, and p-IKKα/β and increased the levels of Bcl-2 and IκB-α. These results suggest that Mor has the potential to combat chronic atrophic gastritis by inhibiting inflammation and apoptosis (Zhang and Wang, 2019).

The protective effects of Mor in colitis are based on its anti-inflammatory activity. In acute colitis mice stimulated by the injection of dextran sodium sulfate, Mor reduced inflammation by decreasing IL-1β, IL-6, and TNF-α expression, improved epithelial defects, and repaired the intestinal mucosal barrier by increasing tight junction proteins (ZO-1, claudin-3, occludins, E-cadherin, and Muc2); additionally, Mor downregulated the overexpression of p-STAT3 and p-p65. In LPS-induced HCT116 cells (a type of human colon cancer cell) and HIEC-6 cells (an epithelial cell from the small intestine), the pro-inflammatory cytokines (IL-1β, IL-6, TNF-α, and IFN-γ) were decreased in the Mor-treated group (Yuan et al., 2020). In the inflammatory bowel disease (IBD) induced by LPS in human colon mucosal epithelial cells (NCM460), Mor reduced apoptosis, inflammation, and oxidative stress by regulating Bax/Bcl-2, inhibiting TNF-α, IL-1β, IL-6, SOD, MDA, T-AOC, and MPO levels. Additionally, Mor suppressed NLRP3 levels and NF-κB pathway activation (Zhang S. et al., 2022).

### 3.7 Protective effect of Mor on the integumentary system

Mor can regulate the hair anagen phase and protect hair follicles to prevent hair loss. In outer root sheath cells, Mor accelerated proliferation by expanding the S and G2 phases and increasing migration, which was partly reversed by DKK1, a Wnt/β-catenin signaling inhibitor. In addition, Mor increased the level of Wnt10b, β-catenin, and lef1. *In vivo*, Mor injection accelerated the onset of anagen and delayed catagen by increasing the diameter of the hair bulbs and skin thickness. In addition, the level of β-catenin was increased in hair follicles (Zhou L. et al., 2018).

Mor can promote wound healing and improve graft healing of the skin. Mor increased the ischemic skin flap survival in rats and elevated flap perfusion by 200%; these effects are related to promoting neovascularization by increasing VEGF and reducing ROS by regulating SOD and MDA (Lin et al., 2020). Mor facilitates the expedited healing of skin wounds by stimulating the proliferation of human epidermal stem cells (EpSCs) and the re-epithelialization of wounds in mice. This is achieved by upregulating β-catenin via GLP-1R-mediated PKA, PKA/PI3K/AKT, and PKA/ERK signaling pathways in EpSCs. Consequently, heightened β-catenin transcriptional activity triggers the transcription of c-Myc, cyclin D1, and cyclin E1, thereby inducing the proliferation of EpSCs (Yu et al., 2024).

In addition, Mor decreased melanin synthesis in human malignant melanoma cells (A375) and keratinocyte co-culture system by inhibiting tyrosinase activity (Yu and Chen, 2013), inhibited A375 proliferation, and promoted apoptosis by downregulating cyclin D1 and Bcl-2 and upregulating P21 and Bax (Li et al., 2017).

### 3.8 Other pharmacological activities of Mor

Other pharmacological effects of Mor are clearly shown in Table 8. In a previous study, *CF* extract inhibited oxidative stress in LPS-induced RAW 264.7 cells (Quah et al., 2020), Mor inhibited the polygonal spindle-shaped pseudopodia and phagocytosis, prevented LPS binding to Toll-like receptor 4 (TLR4), suppressed myeloid differentiation factor 88 (Myd88), reversed the accumulation of NF-κBp65 and degradation of IκB-α, and inhibited pro-inflammatory mediators and cytokines (NO, PGE2, TNF-α, and IL-1β). In addition, Mor inhibited ROS generation by enhancing the expression of Nrf2 and HO-1. Consequently, Mor exerts anti-inflammatory and antioxidant effects by targeting the TLR4/NF-κB and Nrf2/HO-1 signaling pathways (Park C. et al., 2021). In another study, five derivatives of Mor were evaluated in TNF-a-stimulated HUVECs; 7-O-dodecyl morroniside downregulated the expression of E-selectin (Takeda et al., 2010). Moreover, Mor exhibited allergy-preventive effects in mice induced by hen-egg white lysozyme, and the mechanism involves its C-8 position, which possesses the sp^3^ atom, in comparison to compounds ith similar structure (Oku et al., 2011).

**TABLE 8:** Other pharmacological activities of Mor.

Pharmacological activity	Cell or animal model and method	Application	Dose	Molecular mechanism	Reference
Digestive system protection	Chronic atrophic gastritis Wistar rats was established on administration with MNNG combined with irregular diet for 12 weeks	*In vivo*	20, 40, and 60 mg/kg	Gastrointestinal hormones: GAS↓ and MTL ↓ Inflammatory: TNF-α↓, IL-6↓ and IL-1β↓ Apoptosis: Bcl-2↑, Bax↓, cleaved caspase-3↓ and cleaved caspase-9↓ p-NF-κBp65↓, p-IKKα/β↓, IκB-α↑	Zhang and Wang (2019)
	Acute colitis: C57BL/6 mice was established on 2% DSS in drinking water	*In vivo*	90 and 180 mg/kg	Tight junction proteins: ZO-1↑, Claudin-3↑, occluding↑, E-cadherin↑ and Muc2↑ Inflammatory: TNF-α↓, IL-6↓ and IL-1β↓ p-stat3↓ and p-p65↓	Yuan et al. (2020)
	Acute colitis: HCT116 and HIEC-6 cells induced by LPS	*In vitro*	10, 50, and 100 µM	Inflammatory: TNF-α↓, IL-6↓, IL-1β↓ and IFN-γ↓ p-stat3↓ and p-p65↓	
	Inflammatory bowel disease: NCM460 cells induced by LPS	*In vitro*	10, 30, and 60 µM	Inflammatory: TNF-α↓, IL-6↓ and IL-1β↓ Oxidative stress: SOD↑, T-AOC↑ and MDA↓, MPO↓ NLRP3↓, p-p65↓, and p-IκBα↓	Zhang et al. (2022b)
Integumentary system protection epidermal stem cells (EpSCs)	Anagen was induced by depilation in the C57BL/6 mice back skin	*In vivo*	100 μM (intradermally injected)	Skin thickness↑, Bulb diameter↑, Hair cycle score↑	Zhou et al. (2018a)
	Skin flaps SD rat (dorsal random skin flaps, measuring 9 cm in length and 3 cm in width, were elevated from skin and subdermal fat tissue and repositioned)	*In vivo*	30 mg/kg (intraperitoneally injected)	Survival rate↑, the mean blood flow↑ and microvascular density↑ number of VEGF-positive cells↑ SOD↑ and MDA↓	Lin et al. (2020)
	Skin wound C57BL/6 mice (A circular full-thickness skin defect wound was made using a 1.2 cm diameter biopsy punch)	*In vivo*	10, 50, or 100 μg/mL (applied to the wound daily)	The thickness of regenerated epidermis↑	Yu et al. (2024)
	Epidermal stem cells (EpSCs) C57BL/6 mice or from patients pretreated with/without XAV-939, 10058-F4, Ex (9–39), H89, LY294002, or PD98059	*In vitro*	20 μM	EpSC proliferation↑ β-catenin↑, c-Myc↑, cyclin D1↑, and cyclin E1↑ p-AKT↑ and p-ERK↑	
	Co-culture system of human melanocytes and keratinocytes	*In vitro*	0.0001, 0.001, and 0.01 mM	Tyrosinase activity↓ and melanin synthesis↓	Yu and Chen (2013)
	A375 cells	*In vitro*	0.001, 0.01, and 0.1 mM	Apoptosis cells↑, p21↑ and Cyclin D1↓ Bcl-2↓ and Bax↑	Li et al. (2017)
Lung protection	LPS-stimulated rat lung cell lines (E-6TN) and RAW 264.7 cells	*In vitro*	10 μM	IL-6↓, IL-1β↓ and TNF-α↓ p-STAT3↓ p-p65↓	Chen et al. (2022)
	LPS-induced acute lung injury	*In vivo*	90 and 180 mg/kg	Lung W/D↓, MDA↓ and SOD↑ IL-6↓, IL-1β↓ and TNF-α↓ p-STAT3↓ p-p65↓	
	Bleomycin-induced pulmonary fibrosis	*In vivo*	50, 100, and 200 mg/kg	Hydroxyproline↓ Ratio of CD4+/CD8+T cells↓ TGF-β1↓, α-SMA↓ and Collagen I↓	
	Human embryonic lung fibroblast (HELF)-induced by H_2_O_2_	*In vitro*	200 μg/mL	Inhibit apoptosis	Chen et al. (2014)
	Lung cancer A549-induced by H_2_O_2_	*In vitro*	200 μg/mL	No inhibit apoptosis	
Anti-obesity	Adipose-derived stem cells (ADSCs)	*In vitro*	2, 10, and 50 µM	Pparg↓, Cebpa↓, Fabp4↓, Plin2↓, Fasn↓, and Srebp1↓ PPAR gamma↓ and C/EBP alpha↓ Triglyceride content↓ and glycerol↑	Oh et al. (2024)
	Ovariectomized (OVX) obese mice	*In vivo*	2 and 10 mg/kg	Body weight↓ lipid vacuoles in the liver↓, adipose tissue areas↓	
	High-fat diet (HFD)-fed obese mice	*In vivo*	2 and 10 mg/kg	Body weight↓ lipid vacuoles in the liver↓, adipose tissue areas↓	
Eye protection	BALB/c mice were intravitreally injected with 2 μL of LPS to establish a EIU model	*In vivo*	30 and 120 mg/kg	IL-1β↓, IL-6↓, and TNF-α↓ Arg-1↓, iNOS↓, p-JAK2↓, and p-STAT3↓ in ciliary body tissues and retinal tissues	Li et al. (2023b)
	LPS-treated iris pigment epithelial cells (IPE)	*In vitro*	5 μmol/L	Apoptosis cells↓ TNF-α↓, IL-6↓, and IL-8↓ TLR4↓, p-JAK2↓, and p-STAT3↓	Li et al. (2023c)
Reproductive protection	H_2_O_2_-stimulated rat ovarian granulosa cells	*In vitro*	50 mM	ROS↓, MDA↓, SOD↑, GSH-Px↑, and CAT↑ cell apoptosis↓ Caspase-3↓ LC3-II↓, LC3-I↓, beclin-1↓, and p62↓ p-AKT↓, p-mTOR↓	Deng et al. (2021)
	H_2_O_2_-stimulated ovarian granulosa cells	*In vitro*	5, 10, and 20 μM	ROS↓, MDA↓, 8-OHdG↓ and T-AOC↑ SOD↑ and NQO1↑ Apoptosis: Bax↓, Bcl-2↑, cleaved caspase-9↓ and cleaved caspase-3↓ Oxidative stress: nuclear Nrf2↑, p-Nrf2↑, HO -1↑, and NQO1↑	Ma et al. (2022)

Mor has a positive protective effect on the lungs, including acute lung injury, pulmonary fibrosis, and lung cancer. Mor decreased IL-6, IL-1β, and TNF-α levels in LPS-stimulated rat lung cell lines (E-6TN) and RAW 264.7 cells, suggesting its potential to alleviate lung inflammation. Additionally, in mice with LPS-induced acute lung injury, Mor relieved pulmonary edema, oxidative stress, and inflammatory factors such as IL-6, IL-1β, and TNF-α. In mice with bleomycin-induced pulmonary fibrosis, Mor reduced collagen deposition and hydroxyproline accumulation by increasing the CD4+/CD8+ ratio and decreasing the levels of TGF-β1, α-SMA, and type I collagen (Chen et al., 2022). Additionally, Mor has different effects on normal lung cells (human embryonic lung fibroblast, HELF) and lung cancer cells (A549), only in H_2_O_2_-induced HELF Mor promoted cell proliferation, improved cell morphology, inhibited apoptosis, and reduced retinoblastoma protein content, indicating that Mor is a potential ameliorative agent in lung cancer (Chen et al., 2014).

Mor has been found to have an anti-obesity effect. It decreases the expression of genes associated with the formation of fat cells (Pparg, Cebpa, Fabp4, Plin2, Fasn, and Srebp1) in 3T3-L1 cells and ADSCs, which prevents the differentiation of fat cells and the accumulation of fat droplets. This results in lower levels of fat in the cells and a higher release of glycerol. In animal models of obesity (ovary removal (OVX) and HFD-induced obesity), Mor reduced the rate of weight gain and the presence of fat vacuoles and fatty tissue areas (Oh et al., 2024).

Mor has the effect of reducing inflammation in the eye. The human iris pigment epithelium (IPE) plays a vital role in the iris structure. Mor has demonstrated cell proliferation stimulation, apoptosis inhibition, and reduction of levels of inflammatory cytokines such as TNF-α, IL-6, and IL-8 in LPS-IPE cells. In addition, Mor has been observed to suppress the activation of the TLR4/JAK2/STAT3 signaling pathway (Li W. J. et al., 2023). In endotoxin-induced uveitis mice, Mor reduced inflammation by decreasing IL-1β, IL-6, and TNF-α and inhibiting the expression of iNOS, p-JAK2, and p-STAT3 while promoting the expression of Arg-1. This suggests Mor can prevent uveitis inflammation by inhibiting the JAK/STAT pathway to promote M2 polarization (Li W. et al., 2023).

Researchers have also found that Mor regulates and protects follicular development. In H_2_O_2_-induced granulosa cells, Mor reversed survival inhibition and regulated intracellular oxidative stress by decreasing ROS and MDA and increasing SOD, GSH, and CAT. Additionally, Mor inhibited apoptosis by downregulating caspase-3 and autophagy by decreasing LC3-II/LC3-I and beclin-1 levels while increasing p62. Moreover, Mor could activate the PI3K/AKT/mTOR pathway, confirmed by LY294002 and rapamycin (PI3K and mTOR inhibitors) (Deng et al., 2021). In another study, Mor improved the quality of oocytes by reducing oxidative stress in H_2_O_2_-induced ovarian granulosa cells (GCs). Mor decreased the levels of ROS, MDA, and 8-OHdG, upregulated p-Nrf2, and promoted the nuclear translocation of Nrf2, which in turn activated the antioxidants SOD and NQO1. Additionally, Mor reduced apoptosis by regulating Bax, Bcl-2, cleaved caspase-9, and cleaved caspase-3 through the p38 and JNK pathways (Ma et al., 2022).

## 4 Conclusion

According to the traditional medicinal use of *CF*, Mor also demonstrates similar pharmacological effects, and more of its effects have been identified and developed further. The protective effects primarily researched include those on neurodegenerative diseases, focal cerebral ischemia, spinal cord injury, and Alzheimer’s disease. Mor reduces oxidative stress, reduces inflammation, regulates apoptosis, and plays a neuroprotective role primarily via the Nrf2/HO-1 signaling, Bax/Bcl-2, cytochrome C, caspase-3/9, MMP2/3/9, and NF-κB pathways. Mor protects focal cerebral ischemia by promoting angiogenesis and neurogenesis; the former is via Src/Erk, Ang-1/VEGFR signaling and later restrains programmed cell death, oxidative stress, and apoptosis via the Nrf2/HO-1, caspase-3/8/9, and Wnt/β-catenin signaling. Mor prevents Alzheimer’s disease by reducing tau phosphorylation via PI3K/Akt, calpain/GSK-3β, and Src/PP2Ac pathways. In terms of bone and joint protection, the mechanisms of the protective effects on osteoporosis or osteoarticular disease involve regulation of the signaling of caspase-3/9, Bax/Bcl-2, PI3K/Akt/mTOR, Glo1-AGE-RAGE, AKT/Erk, and NF-κB signaling. In addition, the protective effects on the liver and kidney are mediated by regulating lipid metabolism, inflammation, apoptosis, autophagy, and oxidative stress via the AGE/AGER, LXR/ApoE/ABCA1, mTOR, Bax/Bcl-2, caspase-3/9, Nrf2/HO-1, and iNOS/Cox-2 pathways; Mor also promotes hair growth by increasing β-catenin and improves chronic atrophic gastritis and colitis by inhibiting inflammation and apoptosis.; Moreover, Mor increases flap survival, promotes wound healing, and promotes hair growth. Furthermore, Mor improves lung damage, inflammation in the eye, and follicular development. The consistency between the traditional effectiveness of *CF* and Mor suggests the importance of exploring the pharmacodynamic effects according to the theory of TCM. Additionally, the expansion of Mor’s pharmacodynamic effects indicates the need to broaden the scope and treatment directions within TCM.

## 5 Research limitations and perspectives

Mor is the representative iridoid glycoside in *CF*; due to its special structure, Mor cannot be chemically synthesized. However, it can be isolated and purified from *CF*, and the isolation and purification technology is relatively well-developed and straightforward. Given the rapid advancements in polymer materials and their utilization in traditional Chinese medicine chemistry, separating and purifying Mor will soon become simpler and more efficient. In addition, high-resolution mass spectrometry can be utilized to quickly investigate Mor’s plant resources, thereby supporting its development.

Notably, Mor’s structure modification demonstrates diverse pharmacodynamic outcomes. Therefore, conducting a qualitative structure–activity relationship study of Mor is essential for enhancing efficacy, minimizing toxicity, and generating valuable insights for further research.

The study of many pharmacological mechanisms of action of Mor requires more exposure, including its toxicological studies. The therapeutic potential of Mor was explored through existing research and the mechanisms underlying its pharmacological effects. However, the toxicological effect of Mor should be clarified, including chronic and acute toxicities, to guide its clinical use and development better. In addition, we need ongoing high-quality studies to expand the pharmacological effects of Mor, and more comprehensive clinical trials are needed to confirm the long-term efficacy of Mor in human diseases.

The clinical data for Mor are crucial for research as clinical trials are more effective in confirming the safety and efficacy of drugs than cell and animal experiments. Although Chinese herbal formulations such as the Liu Wei Di Huang pill, which is the main ingredient of CF, are commonly used in clinical practice in Asia, there needs to be more separate randomized, double-masked, controlled clinical trials for Mor. Therefore, it is essential to further improve the clinical data to promote its clinical application. Additionally, given the protective effect of Mor on multiple diseases and organs, CF may have more significant medicinal and edible value in future research and utilization.

## Figures and Tables

**Table:** 

**AGEs**	Advanced glycation end products
**BMSC**	Bone marrow mesenchymal stem cell
**CCC**	Countercurrent chromatography
***CF***	Corni Fructus
**CKD**	Chronic kidney disease
**CL**	Clearance
**Cox-2**	Cyclooxygenase-2
**DAD**	Diode array detector
**DN**	Diabetic nephropathy
**ESLD**	Evaporative light-scattering detector
**ESI-MS**	Electrospray ionization mass spectrometry
**EPCs**	Endothelial progenitor cells
**GLP-1**	Glucagon-like peptide-1
**GSH**	Glutathione peroxidase
**GSK-3β**	Glycogen synthase kinase 3β
**HO-1**	Heme oxygenase-1
**IL-10**	Interleukin 10
**MCAO**	Transient occlusion of the middle cerebral artery
**MEKC**	Micellar electrokinetic capillary chromatography
**MDA**	Malondialdehyde
**micro-CT**	Microcomputed tomography
**MMP3**	Matrix metalloproteinase 3
**Mor**	Morroniside
**NF-κB**	Nuclear factor-κB
**NIRS**	Near-infrared spectroscopy
**NLRP3**	Nod-like receptor protein-3
**Nrf2**	Nuclear factor E2-related factor 2
**PGE2**	Prostaglandin E2
**PP2A**	Protein phosphatase 2A
**PS-MS**	Paper spray mass spectrometry
**ROS**	Reactive oxygen species
**SOD**	Superoxide dismutase
**TLR4**	Toll-like receptor 4
**VEGF**	Vascular endothelial growth factor
